# MiR-127-3p targeting CISD1 regulates autophagy in hypoxic–ischemic cortex

**DOI:** 10.1038/s41419-021-03541-x

**Published:** 2021-03-15

**Authors:** Zi-Bin Zhang, Liu-Lin Xiong, Lu-Lu Xue, Yan-Ping Deng, Ruo-Lan Du, Qiao Hu, Yang Xu, Si-Jin Yang, Ting-Hua Wang

**Affiliations:** 1 Institute of Neurological Disease, Translational Neuroscience Center, West China Hospital, Sichuan University, Chengdu, 610041 China; 2 Department of Anesthesiology, Qilu Hospital, Cheeloo College of Medicine, Shandong University, Jinan, Shandong Province 251102 China; 3 Animal Zoology Department, Institute of Neuroscience, Kunming Medical University, Kunming, Yunnan 650500 China; 4 National Traditional Chinese Medicine Clinical Research Base and Western Medicine Translational Medicine Research Center, Department of Anaesthesiology, Affiliated Traditional Chinese Medicine Hospital, Southwest Medical University, Luzhou, 646000 Sichuan China

**Keywords:** Autophagy, Molecular biology

## Abstract

Neonatal hypoxic–ischemic (HI) injury derived from asphyxia during perinatal period, is a serious complication of neonatal asphyxia and the main cause of neonatal acute death and chronic neurological injury. Aberrant autophagy occurs in many nervous system diseases, but its role and underlying mechanism in HI injury is largely unknown. Here, we successfully constructed a newborn rat model of HI brain injury, and the knockout-miR-127-3p (KO-miR-127-3p) rats were structured by using CRISPR/Cas9. Subsequently, the in vitro functional experiments, in vivo zea-longa scores, as well as bioinformatics analyses and biological experiments were applied. The expression of autophagy-related proteins, including ATG12, P62, Beclin-1, LC3II in HI cortex with miR-127-3p knockout was significantly decreased, and autophagic vacuoles were disappeared. Moreover, miR-127-3p has a specific regulatory effect on CISD1 expression, another crucial molecule in autophagy process. Accordingly, the overexpression of CISD1 effectively inhibited the autophagic cell death and physiological dysfunction in the brain of HI injury, whereas si-CISD1 reversed the neuroprotective effects of KO-miR-127-3p. Our findings explained the underlying mechanism for HI injury, and miR-127-3p targeting CISD1 signal could be supposed as a new treatment strategy to prevent and treat HI injury.

## Introduction

Neonatal hypoxia–ischemia (HI) injury caused by hypoxia, cerebral blood flow reduction, or short-term cerebral blood flow perfusion interruption in perinatal asphyxia, is a severe disease that usually results in a series of clinical symptoms and signs of central nervous abnormalities. HI injury in brain is becoming one of the important causes of neonatal death in acute perinatal period and long-term neurological sequelae in the clinic^1,2^, which has endangered the life quality of children and brought enormous economic burden to the country and society. The mechanisms of HI have been reported to include hemodynamic change, oxidative stress, calcium influx, energy metabolic disorder, excitotoxicity, and cell death. Among them, the forms of neuronal cell death processes consisting of necrosis, apoptosis, and autophagy are very important pathological process^3^, of which neuronal autophagy plays an important role in the regulation of homeostasis and protein functions^4^. Of these, the increased expression of Beclin-1 can be used as a marker of autophagy in neonatal rats; thus, autophagy of neurons after HI could be observed. Meanwhile, a potential protective mechanism in the early stage of HI brain injury may be the overactivation of autophagic pathway^5^. Therefore, autophagy, designated as a general term for the degradation of intracellular substance by lysosomal pathway, is defined as mainly responsible for the degradation and reuse of long-lived protein and organelles, and it is characterized by a large number of bilayer membrane structures under the electron microscope^6,7^. The untimely occurrence or excessive activation of autophagy can definitely trigger Type II programmed cell death that is unlike cell apoptosis. Moreover, Type II programmed cell death caused by autophagy may be involved in regulating neuronal death caused by ischemia^8^. Therefore, it is of great scientific significance and clinical application value to reveal the regulating mechanism for autophagy in neonatal HI brain injury.

MicroRNAs (miRNAs), known as types of endogenous small noncoding RNA molecules, ranging in length from 4 to 24 nucleotides, play important roles in pathophysiological regulation of central nervous system (CNS), such as effecting the nerve regeneration, apoptotic necrosis, and mitochondrial damage.^9,10^. In order to achieve neuroprotective goals, microRNA-210 was inhibited in neonatal rats with HI brain injury^11^. Moreover, miRNAs may participate in regulating autophagy through their effects on various autophagy regulatory proteins, which not only play key roles at various steps of the autophagy pathway, but also participate in signaling pathways to later autolysosome degradation^12^. While autophagy plays a fundamental role in the development of the central nervous system^13^. However, the mechanism of autophagy regulating by microRNA in neonatal HI is waiting to be elucidated. The miR-127 family, including miR-127-5p and miR-127-3p, is one of the miRNAs that located within an miRNA cluster in the Dlk1–Dio3 region. In recent years, it has been found that miR-127-3p could inhibit the proliferation and invasion of human osteosarcoma cells by targeting ITGA6 or SETD8 (ref. ^14,15^), the proliferation and differentiation of myoblasts regulated by reciprocation were achieved by targeting Vamp2 directly^16^. Meanwhile, miR-127-3p could help to diagnose FTD and to distinguish it from AD^17^. In addition, miR-127-3p targeted KIF3B through suppressing cell invasion, migration, and proliferation can inhibit the development of OSCC^18^, indicating that miR-127-3p is important in several biological processes. However, the effect of miR-127-3p in neonatal HI injury of brain is largely unknown, and the mechanism of miR-127-3p- associated CDGSH iron–sulfur domain-containing protein 1 (CISD1) to regulate autophagic neuron death, is completely unknown, and is needing to be revealed.

This study was designed to evaluate the function of miR-127-3p in the autophagic program of neonatal HI injury and provide new ideas for the diagnosis and treatment of neonatal HI. By using miRNA sequencing combined with experimental validation, miR-127-3p was found to downregulate at 12 h (h) and upregulate at 48 h and 96 h after HI in the cerebral cortex of neonatal rats. In order to verify the function of miR-127-3p, the knockout miR-127-3p (KO-miR-127-3p) rats were structured by CRISPR/Cas9, and the suppression of autophagic cell death via KO-miR-127-3p as well as the physiological dysfunction in the neonatal HI injury were confirmed. Afterward, bioinformatics analyses and biological experiments manifested that miR-127-3p has specific modulation for the expression of CISD1. Moreover, it was affirmed that the overexpression of CISD1 effectively inhibited the autophagic cell death and physiological dysfunction in the brain of neonatal HI injury, whereas si-CISD1 reversed the neuroprotective effects of KO-miR-127-3p. Our data, therefore, demonstrated that the interaction of miR-127-3p regulating CISD1 improved autophagic program in neonatal HI injury, and it could therefore provide a new candidate target for the therapy of neonatal HI in future clinic practice.

## Materials and methods

### Regents

Anti-Tuj1 rabbit antibody (1:200, Abcam, UK) and the fluorescein-conjugated secondary antibody (1:800, Abcam, UK). Anti-Beclin-1 rabbit antibody (1:2000 ABclonal, USA), Anti-LC3I rabbit antibody (1:2000, ABclonal, USA), Anti-LC3II rabbit antibody (1:2000, ABclonal, USA), Anti-P62 mouse antibody (1:2000, ABclonal, USA), Anti-ATG12 mouse antibody (1:2000, ABclonal, USA), and secondary antibody (goat anti-rabbit IgG and goat anti-mouse IgG; ZSGB-BIO, Beijing, China, 1:5000)

### Animal care and grouping

Seven-day-old (7-d-old) Sprague-Dawley (SD) rats (both male and female, weighting at 10–19 g) were provided by Animal Zoology Department, Kunming Medical University. All the rats were allowed to eat and drink freely in a 12-h light/dark cycle at 21–25 °C with the humidity of 45–50%. All experimental procedures were carried out in accordance with the procedures approved by the Animal Care Committee, Kunming Medical University. Feeding and nursing of laboratory animals followed the rules of the China Laboratory Animal Protection and Ethics Committee, as well as in line with the National Institute of Health and Health Laboratory Animal Ethics Guide. Sample size was estimated from the sum of the minimum sample sizes required for each experiment. All experimental animals were grouped using a simple random sampling method. All the measurements and observations were performed by researchers who were blinded to group allocation and repeated three times. Both the number of samples and animals used in this experiment were used for analysis.

### Neonatal HI injury models

Neonatal HI injury models in rats were established and referred to the classical Rice–Vannucci method^19^. Briefly, after anesthetized with isoflurane, rats were disinfected with iodine, and followed with the longitudinal incision (about 1 cm) in the center of the neck to expose the right common carotid artery (CCA). Then blood vessels were blocked by electrocoagulation, and the subcutaneous tissue and skin were stitched. Last, the rats were taken back to the mother to restore for 1 h. Afterward, the postoperative neonatal rats were been put in the plexiglass anoxic tank with a length of 35 cm, a width of 15 cm, and a height of 20 cm. Anoxic gas (8% oxygen and 92% nitrogen gas mixture) was led into the tank for 2 h, with controlling the flow rate of 1–2 L/min. The airtight anoxic bottom part required to place on 37 °C electric blanket to keep warm. The rats in sham group suffered the same anesthesia and arterial exposure, without the arterial occlusion and hypoxia.

### Tissue harvest

After rats were anesthetized by inhaling isoflurane, their chest was opened and the perfusion tube was fixed from the apical part of the perfusion tube to the ascending aorta. Then, the 15-ml phosphate-buffered saline (PBS) was quickly poured to clean the blood. Last, the brain tissue was acquired and put into the −80 °C refrigerator for preservation.

### 2,3,5-Triphenyltetrazolium hydrochloride (TTC) staining

TTC staining was performed according to a protocol adapted from Van Waes Debergh. The rats were anesthetized with isoflurane inhalation at 72 h post HI, and then the head of rats were obtained. Subsequently, the whole-brain tissue was frozen at −20 °C for 10 min and evenly cut into 5 slices on the coronal plane, and incubated with 2% TTC solution for 30 min at 37 °C. The brain tissue was fixed with 4% polyformaldehyde for 24 h after staining. The images of slices were captured on the next day and the infarct area of the slice was measured by Image analysis software ImagePro Plus. The volume of cerebral infarction was calculated according to the area of brain slices and the distance between the slices. Infarct volumes were calculated by indirect method: cerebral infarction volume/total cerebral volume × 100%.

### MicroRNAs high-throughput screening (HTS)

HTS was carried out in 2 rats (one biological repeat) in sham group and 2 rats in HI group with the highest small RNA abundance. According to the manufacturer’s protocol, the small RNA library was constructed by TruSeq Small RNA sample preparation kit (Illumina, San Diego, CA). In brief, the small RNA samples (5–10 ng) were connected with 59 and 39 adapters, and then reverse transcription-polymerase chain reaction (RT-PCR) was carried out to construct the cDNA library and index the tags. The cDNA library fragments were purified and separated by 6% TBE PAGE gel, and miRNA inserts was obtained. The samples of cDNA library were collected in equal molarity, and were clustered and sequenced in a single lane on the platform of Illumina NHISeq2500 platform (50-bp single read).

### Quantitative reverse transcription polymerase chain reaction (qRT-PCR)

Total RNA was extracted from cerebral cortices by Trizol reagent (TaKaRa, Japan) according to the manufacturer’s protocol, and was then reverse-transcribed to complementary DNA (cDNA) according to the instruction of Revert Aid^TM^ First Strand cDNA Synthesis kit (Thermoscietific). Subsequently, the qRT-PCR was performed in a thermal cycler (CFX96); β-actin and U6 were used as an internal reference. The primer sequences are listed in Table 1. Then, the relative expression of mRNAs was performed by qRT-PCR. The reaction conditions were initial stage, 95 °C for 10 min; cycle stage, denaturation, 95 °C for 15 s (s); annealing and extension, 60 °C for 60 s.

**Table 1:** Primer sequences.

Gene	Forward (5’-3’)	Reverse (5’-3’)
MiR-127-3p	CTTATCGGATCCGTCTGAGC	CAGTGCAGGGTCCGAGGTAT
CISD1	GCTCTCGGTTACCTGGCTTA	TTGTCTCCAGTCTCCTCATTGT
KIF3B	ATTCAGCAGCAGATGGAGAGT	CTTGTATCCTACGGCAGAGACT
U6	GCTTCGGCAGCACATATACTAA	AACGCTTCACGAATTTGCGT
β-actin	GAAGATCAAGATCATTGCTCCT	TACTCCTGCTTGCTGATCCA

### Culture of PC12 cells and SY5Y cells

PC12 cell line (GCC-KI0009RT) was obtained from Shanghai Genechem Co., LTD and SY5Y cell line (BFN60700126) was obtained from BLUEFBIO. Neither cell line is contaminated with HIV-1, HBV, HCV, mycoplasma, bacteria, yeast, and fungi. The frozen PC12 cells and SY5Y cells were taken from the −150 °C refrigerator and quickly melted into a 37 °C water bath according to the routine method, then plated at a density of 5 × 10^5^ cells/ml in 25-cm^2^ culture flasks to move into the CO_2_ hatch and start to cultivate. PC12 cells and SY5Y cells were passaged after seeding for 2 d, and then used for experimentation.

### Culture of primary cortical neurons

The culture of primary cortical neurons was carried out as previously described^20^. Briefly, the cortical tissue of 1-day-old SD rats was harvested, minced, and isolated with 0.25% trypsin for 10–15 min at 37 °C. The samples were then eluted by 10% fetal bovine serum, and centrifuged (1500 rpm for 5 min) at room temperature. After centrifugation, the supernatant was discarded, and fresh complete culture medium was used for preparation of single-cell suspension. Subsequently, with about 2–5 × 10^5^/ml density, the cells were seeded on the poly-L-lysine and laminin coated cover glasses and inoculated with 5% CO_2_ and 95% air at 37 °C. Moreover, the complete medium was replaced by neuron specific medium (neuron basal + 2% B27, no serum) (Gibco, USA). The culture medium was changed every 3 days.

### Establishment of oxygen glucose deprivation (OGD) model

OGD model was established on primary cultured neurons to mimic HI condition in vitro. According to previous researchers^21–23^, glucose-free DMEM medium is a great substitute for the cell culture media, and the neurons, PC12 cells and SY5Y cells in the culture plate were washed three times with PBS and then incubated with glucose-free DMEM medium. Then the cells were placed into a hypoxia chamber with 0% oxygen, 95% nitrogen, and 5% carbon dioxide at 37 °C. After hypoxia (1 h for neurons and SY5Y, 6 h for PC12), the glucose-free DMEM medium was replaced by the normal DMEM medium. Cells were finally placed in the cell incubator containing 95% air and 5% CO_2_ at 37 °C for 24 h.

### Transfection of miR-127-3p mimic and inhibitor into cells

PC12 cells and SY5Y cells were cultured for 1 day, and cortical neurons were cultured for 3 d until the cells grow at about 50–80% confluence prior to transfection. For transfection, normal group, OGD group, mimic-NC group, anti-NC group, miR-127-3p group, and anti-miR-127-3p group. MiR-127-3p mimic and anti-miR-127-3p were designed were synthesized by RiboBio (Guangzhou, China). Super Fectin TM II in vitro transfection reagent (Pufei Biotech, China) was applied in transfection. In brief, 3 μl SuperFectin™ II reagent was added to the prepared mixture of transfected stock buffer and miRNA. Mixtures of miR-127 mimics (50 nm) and anti-mir-127 (100 nm) were added to the appropriate pore drop by drop.

### Cell viability assay (CCK8)

Cell counting Kit-8 (CCK8, Dojindo, Kumamoto, Japan) measures cell viability based on the manufacturer’s introduction. Neurons were plated in 96-well plates at each required density with Neurobasal®-A medium containing 2% B27 for 3 d. After HI for 24 h, CCK8 solution was added to each plate and then incubated at 37 °C for 3 h. The optical densities (OD) value of each group was detected at 450 nm.

### TUJ1 staining

The neurons were rinsed once with PBS, then fixed with paraformaldehyde for 15 min, and finally rinsed with PBS for 3 times, each time for 5 min, so as to achieve the purpose of clearing the fixed solution. Anti-Tuj1 rabbit antibody (1:200, Abcam, UK) was then added. Next, the neurons were incubated overnight at 4 °C, washed with PBS for 3 times, each time for 5 min. Then, the fluorescein-conjugated secondary antibody (1:800, Abcam, UK) was added into the neurons to incubate for 1.5 h at 37 °C. The neurons were washed with PBS for three times (5 min per time). Finally, the neurons were stained by DAPI (Invitrogen, USA).

### Reactive oxygen species test

To test active oxygen level in Neuronal with 24 h OGD, fluorescent probe DCFH-DA (ROS Assay kit, Blue Sky Company, China) was used for each group of cultured neurons. We diluted DCFH-DA (1:1000) with serum-free neurobasal medium (final concentration 10 micro mole/L), then discard the supernatant, and add the serum-free high-glucose DMEM culture liquid to clean two times. Next, we added appropriate volume to dilute DCFH-DA. Cells were incubated at 37 °C for 20 min, and the old culture medium was discarded to wash the cells by no-serum high-glucose DMEM three times. Last, under inverted fluorescence microscope (the 488-nm excitation wavelength and 525-nm emission wavelength), cell fluorescence was observed, the conditions of cells that produced ROS were detected, and the results were collected.

### Mito tracker test

First, the Mito-tracker green storage liquid was prepared. The Mito-tracker green was prepared to the concentration of 1 mM with anhydrous DMSO (dimethylsulfoxide). Again, the Mito-tracker green working fluid was prepared. A small amount of 1 mM Mito-tracker Green storage liquid was added to the serum-free neurobasal medium according to the 1:5000–1:50,000 proportion, so that the final concentration was 20–200 nm. After blending, it was Mito-TrackerGreen working fluid. Third, after 24 h of neuronal glucose deprivation, the mitochondrial fluorescence markers were performed on the cultured neurons of each group. Removing the neuron culture solution, cells were added at 37 °C Mito-tracker Green dye working fluid, and incubated for 30 min in the incubator. Finally, the Mito-tracker green staining working fluid was removed, cells were added 37 °C neurobasal medium, and observed the mitochondrial fluorescence staining with fluorescence microscope.

### Terminal-deoxynucleotidyl transferase-mediated nick-end labeling (TUNEL) staining

To assess DNA damage, neurons were prepared and the apoptosis was detected by TUNEL assay kit (Roche, Germany). The staining was executed in accordance with the manufacturers’ instruction. The positive cells exhibited red staining within the nucleus of apoptotic cells. The total number of TUNEL-positive neurons was calculated in three different regions for each section. The image was obtained by fluorescence microscope.

### miR-127-3p-knockout SD rats

MiR-127-3p-knockout (miR-127-3p−/−) rats were purchased from Cyagen Biosciences Inc., USA. MiR-127-3p+ /− rats were interbred to give miR-127-3p−/− rats, and the rats were genotyped by multiplex PCR (Cyagen Biosciences Inc, USA). Rat miR-127-3p F: 5′-TCATTGTATAGGGCCGGTAGCATCTC-3′, R: 5′-TGTCCACCAGAAGCACTA TGAGTGGA-3′. All experiments were performed on miR-127-3p−/− rats and their wild-type (WT) littermates (miR-127-3p+/+).

### Western blot (WB)

In order to detect proteins related to ischemic brain injury, western blot was used. Briefly, 400 μg of total protein was extracted and dissolved by 15% SDS-polyacrylamide gel. Afterward, the protein was separated to protein bands at 80 voltage (V) sustaining 20 min and then 120 V sustaining 30 min by using the electrophoresis buffer (24.8 mM Tris, 192 mM glycine, and 0.1% SDS). Then, the transfer buffer (24.8 mM Tris, 192 mM glycine, and 10% methanol) was added, the protein on the SDS-PAGE gel was transferred to PVDF membranes for 4 h at a rate of 350 mA. The PVDF membranes were blocked by 5% skim milk at room temperature for 1–2 h, and were then incubated with primary antibody (Beclin-1, 1:2000, ABclonal, USA; LC3I, 1:2000, ABclonal, USA; LC3II, 1:2000, ABclonal, USA, P62, 1: 2000, ABclonal, USA; ATG12, 1:2000, ABclonal, USA) in 3% BSA overnight at 4 °C. After washing in TBST 3 times for 8 min each time, the PVDF was incubated in the secondary antibody (goat anti-rabbit IgG and goat anti-mouse IgG; ZSGB-BIO, Beijing, China, 1:5000) for 1~2 h at room temperature. Finally, the PVDF membranes were rinsed in TBST three times to get ECL luminescence, scanning, and image acquisition using Alpha Ease FC Gel Imaging System (BIO-RAD, USA) with ECL.

### Grip test

Grip test was used to test the neurological function and fatigue of rats after hypoxic ischemic injury^24^. The experiment was performed on rats at 1st day, 3rd day, and 7th day after the operation. The neonatal rats were holding the metal ropes (2 mm in diameter) on both sides of the front foot, and the wires were stretched horizontally in a test box (high at 25 cm inside the box) with a width of 50 cm fixed at both ends. We prepared the bottle of the box covered with soft cork dust. The time of the rats from holding the metal rope to releasing the metal rope was recorded in seconds three times to take the maximum time to include the score. All trials were performed in a double-blind state of two participants.

### Rotarod test

The experiment was employed to investigate the coordination ability of long-term exercise in rats after hypoxic–ischemic brain injury. The test instrument was provided by Shanghai Xin Soft Information Technology Co., Ltd. (model: XR1514). Rats were tested at 38 days after hypoxic–ischemic brain injury. Besides, rats in each group were trained in adaptability 3 days before the experiment. In formal trials, the groups of rats were placed on the rotarod, the rods were accelerated from 4 revolutions per minute (RPM) to 40 RMP in 3 min, followed by 40 RMP recording the time of the rat from the stick to the drop, which were measured in seconds and three consecutive times. Finally, the maximum time was included in the score. All trials were performed in a double-blind state of three participants.

### Construction of the viral vector to determine the role of CISD1 in HI model

To explore the effect of CISD1 in NHI, lentivirus-based vectors were applied. The plasmid, Genechem Co., Ltd., China provides primers and restriction endonucleases for our experiments. In the overexpression of CISD1, the cDNA of CISD1 sequences was obtained through qRT-PCR. Ubi-F 5′-GGGTCAATATGTAATTTTCAGTG-3′, FLAG-R-2 5′-CCTTATAGTCCTTATCATCGTC-3′. The rat CISD1 RNA interference (siRNA) target sequence is 5′-GCACCAAAGCTATGGTGAA-3′. A scramble form was used as a control, 5′-TTCTCCGAACGTGTCACGT-3′. All constructs were amplified in HEK293 cells, which were cultured in DMEM containing 10% FBS (Invitrogen, USA).

### Luciferase activity assays

The luciferase activity assays were performed as previously descried^25^. CISD1 3′ UTR luciferase plasmids and the corresponding mutant plasmids were produced by RiboBio (Guang Zhou, China). More details can be found in our previously published work^26^.

### Data collection

The result of TTC staining, immunohistochemistry, immunofluorescence, and western blot were managed and obtained by using image J and IPP software. QPCR results are expressed by relative quantitative analysis *F* = 2−^∆∆Ct^, ^∆Ct^ = Ct value of target gene-Ct value of internal reference gene; −^∆∆Ct^ = average value of NC group ∆Ct-value of each sample ∆Ct. 2^−∆∆Ct^ reflects the relative expression quantity of the target gene of each sample relative to the NC group.

### Statistical analysis

SPSS 20.0 Statistical software is used for data statistics. The results were indicated by the mean ± standard deviation (s.d.). *T*-test was used for comparison between the two groups, and the data were analyzed by one-way ANOVA analysis of variance, when there were three or more groups. The Kruskal–Wallis test was used for multiple-group comparisons that did not conform to normal homogeneity and homogeneity of variance. *P* < 0.05 was considered statistically significant.

## Results

### The specific expression of miR-127-3p in neonatal HI injury

**Fig. 1:**
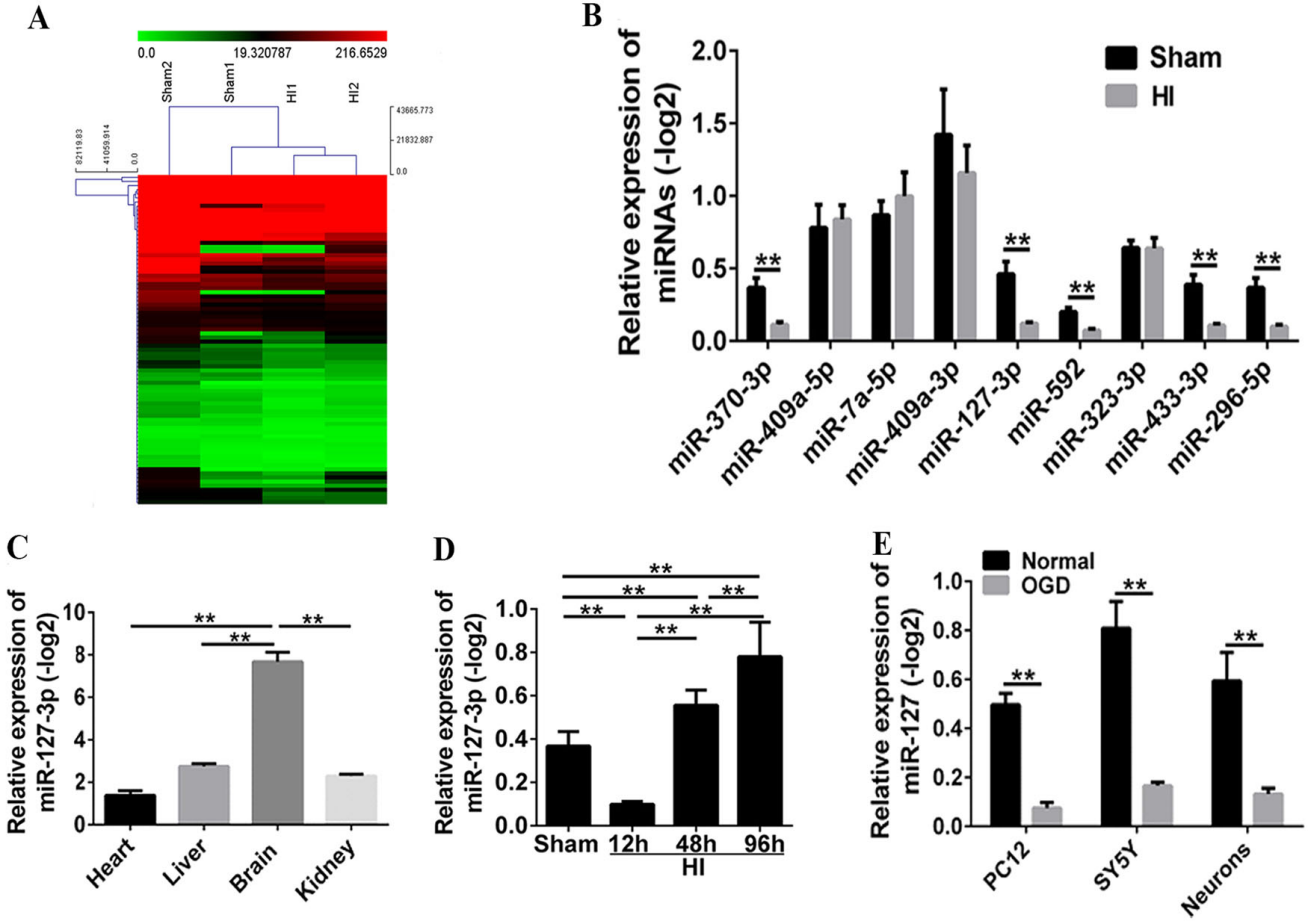
The specific expression of miR-127-3p in vivo and in vitro. **A** Results of miRNA sequencing analysis showed that the expression of miRNAs in sham and HI have obvious difference (*n* = 2/group). **B** The qRT-PCR validation of differential miRNAs in normal group rats and HI group rats at 12 h after HI injury. Each bar was the mean ± s.d. relative to −log2 (**P < 0.01 with Student’s *t*-test, *n* = 6). **C** Relative expression of miRNA-127-3p in heart, liver, brain, and kidney of rats. MiRNA-127-3p was selectively highly expressed in the brain (**P < 0.01 with one-way ANOVA, *n* = 6). **D** qRT-PCR analysis of miR-127-3p expression at 12, 48, and 96 h. MiRNA-127-3p decreased at 12 h after HI, while it increased at 48 and 96 h after HI injury (**P < 0.01 with one-way ANOVA, *n* = 6). **E** The relative expression of miR-127-3p in OGD of PC12, SY5Y, and neurons was significantly decreased in vitro (**P < 0.01 with Student’s *t*-test, *n* = 6). HI hypoxic ischemic.

To explore the potential effect of miR-127-3p on the prevention and treatment of neonatal HI injury, the HI model of new-born rat was successfully constructed. The results of miRNA sequencing analysis showed that the expression of miRNAs between sham and HI group exhibited obvious differences (Fig. 1A). Of these, the expressions on the miR-370-3p, miR-127-3p, miR-592, miR-433-3p, and miR-296-5p were downregulated after neonatal HI (versus sham group) (Fig. 1B, *P* < 0.01), and miR-127-3p was one of the most significantly downregulated miRNAs. Besides, the results of qRT-PCR in different organs showed that miR-127-3p was specifically and highly expressed in the brain tissue from normal neonatal SD rats, which indicated that miR-127-3p was a miRNA specifically expressed in the central nervous system (Fig. 1C, *P* < 0.01); it decreased at 12 h, followed by increased at 48 h and 96 h after HI (Fig. 1D, *P* < 0.01). Moreover, the relative expression of miR-127-3p in OGD of PC12, SY5Y, and neurons was significantly decreased in vitro (Fig. 1E, *P* < 0.01).

### Sequestration of miR-127-3p alleviated mitochondrial and neuron injury induced by OGD in vitro

**Fig. 2:**
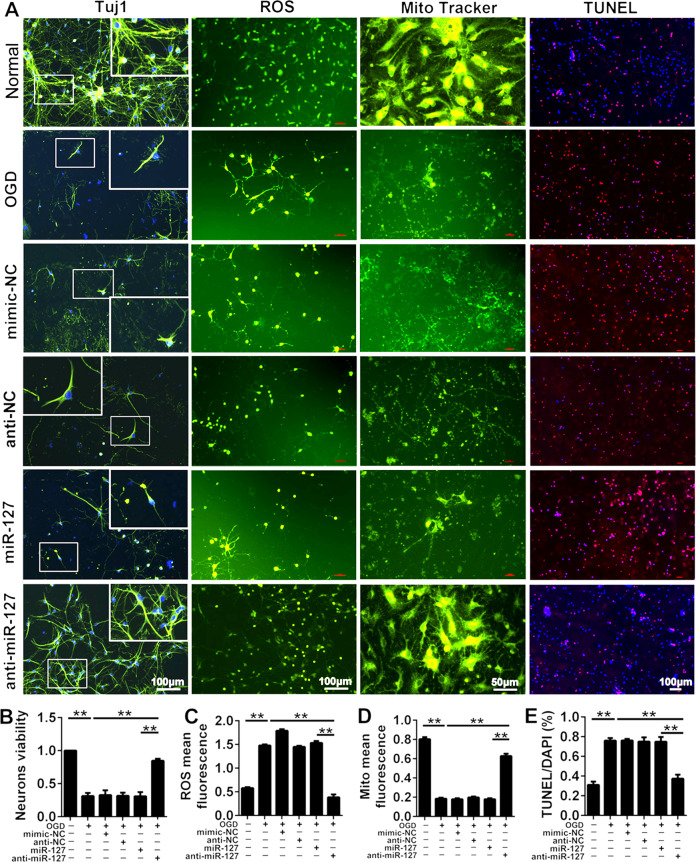
The effects of sequestration of miR-127-3p on mitochondrial function and neuron injury induced by OGD in vitro. **A** Neurons were stained by Tuj1, reactive oxygen species (ROS), Mito tracker and TUNEL to detect the condition of neurons, the level of ROS, the condition of mitochondria and neurons apoptosis in normal group, OGD group, mimic-NC group, anti-NC group, miR-127-3-p group, and anti-miR-127-3p group. Scale bar = 100 μm (Mito tracker: scale bar = 50 μm). **B** The bar charts of neurons viability in neurons of each group. Compared with normal group, the neurons viability decreased in OGD group, there was statistic significant. While the neurons viability increased in anti-miR-127-3p group, compared with OGD group. **C** The level of ROS in neurons of each group. ROS significantly increased in OGD group, compared with normal group. Whereas, ROS were significantly decreased in anti-miR-127-3p group compared with OGD group. **D** The mitochondria mean fluorescence in neurons of each group. Mito mean fluorescence significantly decreased in OGD group. Whereas mito mean fluorescence significantly increased in anti-miR-127-3p group, compared with OGD group. **E** Percentage of TUNEL/DAPI of neurons was shown in each group. TUNEL staining was used to analyze neuronal apoptosis. The percentage of TUNEL/DAPI significantly increased in OGD group, compared with normal group. Whereas the percentage of TUNEL/DAPI significantly decreased in anti-miR-127-3p group, compared with OGD group. The data were presented as the means ± s.d. **P < 0.01 with one-way ANOVA or Kruskal–Wallis, *n* = 6. OGD oxygen glucose deprivation, NC negative control, HI hypoxic ischemic, Mir microRNA, TUNEL terminal deoxynucleotidyl transferase dUTP nick-end labeling, ROS reactive oxygen species, DAPI 2-(4-amidinophenyl)-6-indolecarbamidine dihydrochloride.

To explore the role of miR-127-3p in the cerebral cortex of HI-injured newborn rats, PC12 cells, SY5Y cells, and neurons were transfected by miR-127-3p mimic and miR-127-3p inhibitor, respectively. The number of the three types of cells have reduced significantly after OGD at 24 h, while the cells number in anti-miR-127-3p group were increased compared with OGD group (Fig. S1, *P* < 0.005). However, there is no significant difference in cell number between miR-127-3p and OGD groups. The results suggested inhibition of miR-127-3p markedly improved the cell injury caused by hypoxia. Furthermore, the neuroprotection of miR-127-3p inhibitor in cortical neurons was investigated by detecting the morphology of cortical neurons, the level of reactive oxygen species (ROS), mitochondrial activity, and cell apoptosis in OGD cortical neurons (Fig. 2A). The result of cell viability assay showed that the suppression of miR-127-3p greatly ameliorated the vitality of cortical neurons in OGD (Fig. 2B, *P* < 0.01). Meanwhile, hypoxia in cortical neurons markedly induced the increase of ROS, impeded mitochondrial and neuronal activity, and increased cortical neurons death. Whereas, transfection of miR-127-3p inhibitor manifested a significant decrease of ROS (Fig. 2C, *P* < 0.01), improvement of mitochondrial activity (Fig. 2D, *P* < 0.01), and decrease of the apoptosis number of cortical neurons (Fig. 2E, *P* < 0.005). As a consequence, the neuroprotective effect on the inhibition of miR-127-3p was apparent, which is associated with inhibiting the generation of ROS and the improvement of mitochondrial activity.

### KO-miR-127-3p relieved autophagy and neurological impairment in vivo

**Fig. 3:**
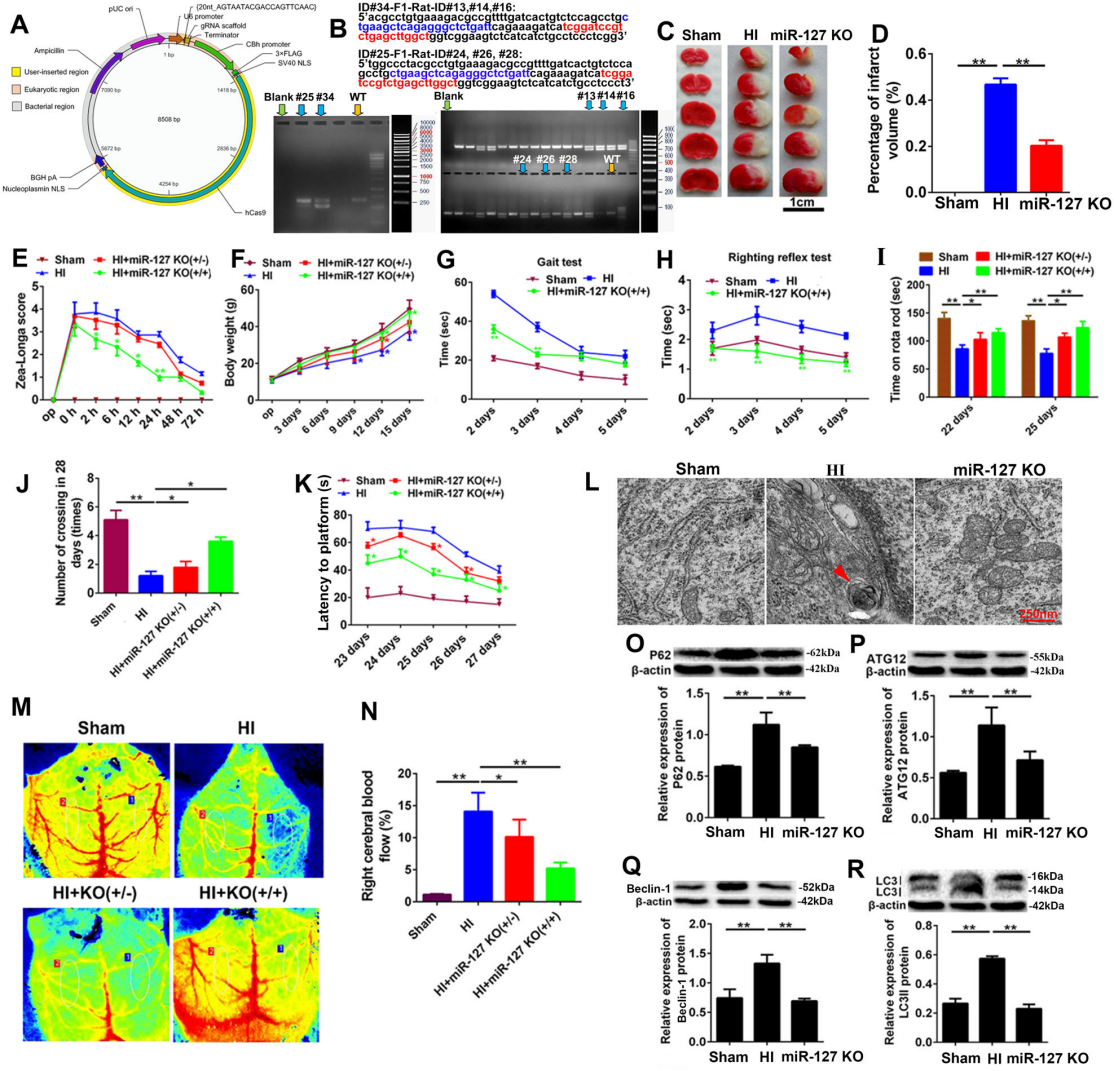
KO-miR-127-3p relieved autophagy and neurological impairment in vivo. **A** Vector construction drawing of KO-miR-127-3p rats. **B** The examination of miR-127-3p expression in KO-miR-127-3p. F0 founders PCR screening: the molecular weight of wild type is 677 bp, # 25 missing 133 bp, # 34 missing 129 bp. F1 founders PCR screening: the molecular weight of wild type is 677 bp, # 24, 26 #, # 28 missing 133 bp, # 13, # 14, # 16 missing 129 bp. **C**, **D** TTC staining of rat brains. Images above the graph show representative slices from each group. Brains were sliced, stained with TTC, and fixed to delineate live (red) from dead or infarcted tissue (white). Infarcts were quantified by planimetry and expressed as a percentage of risk zones. Scale bar =1 cm. **E** Zea-longa score of rats. **F** Results of body weight. **G**, **H** Grip times and righting reflex time were assessed in 2 d, 3 d, 4 d, and 5 d after HI. **I** Time on rota rod was assessed in 22 d and 25 d after HI. **J** Number of crossing in 28 days. **K** Latency to plant forms. **L** Representative EM images of autophagic vacuoles were shown. Scale bar = 250 nm. The arrows depict autophagosomes. **M**, **N** Right cerebral blood flow of HI rats. **O**, **P**–**R** The levels of P62, ATG12, Beclin-1, LC3 I, and LC3II were detected by WB in Sham group, HI group and KO-miR-127-3p group. Data are shown as mean ± s.d. **P < 0.01 with one-way ANOVA or Kruskal–Wallis, *n* = 6. HI hypoxic ischemic, KO knock out, WB western blot, TTC 2,3,5-triphenyl-2H-tetrazolium chloride, D days, WT wild type, Mut mutant, NC negative control, H hours.

**Fig. 4:**
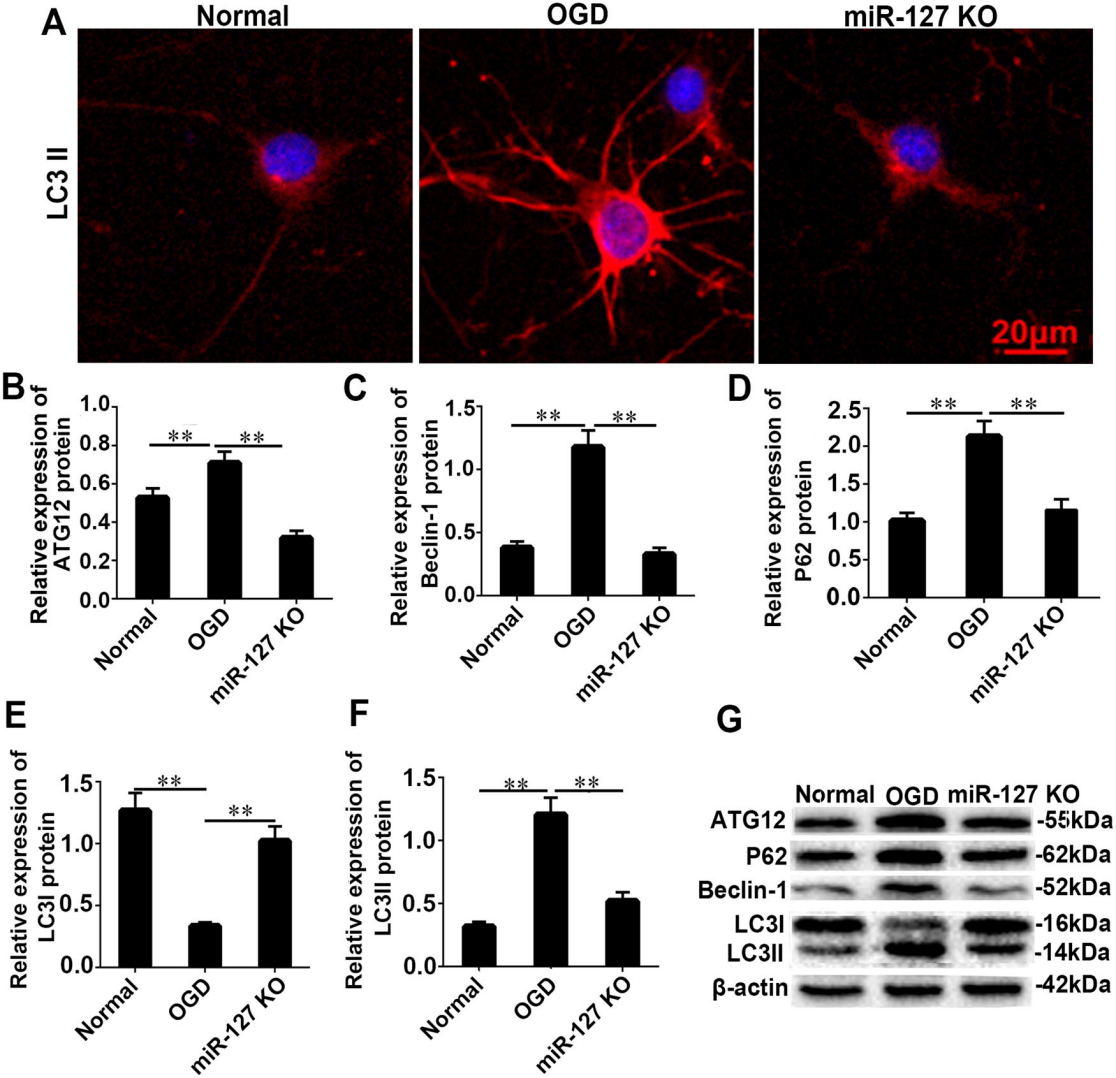
KO-miR-127-3p relieved autophagy in vitro. **A** Autophagy of neurons was increased significantly in OGD, and reduced significantly in miR-127-3p inhibitor. **B**, **C**–**G** The relative expression of ATG12, Beclin-1, P62, and LC 3II was obviously increased, and the relative expression of LC 3I was decreased significantly. However, the expression result of ATG12, Beclin-1, P62, LC3I, and LC3II was opposite in the miR127 KO rat group. OGD oxygen glucose deprivation, KO knock out. Scale bar = 20 μm. The data are presented as the means ± s.d. **P < 0.01 with one-way ANOVA, *n* = 6.

**Fig. 5:**
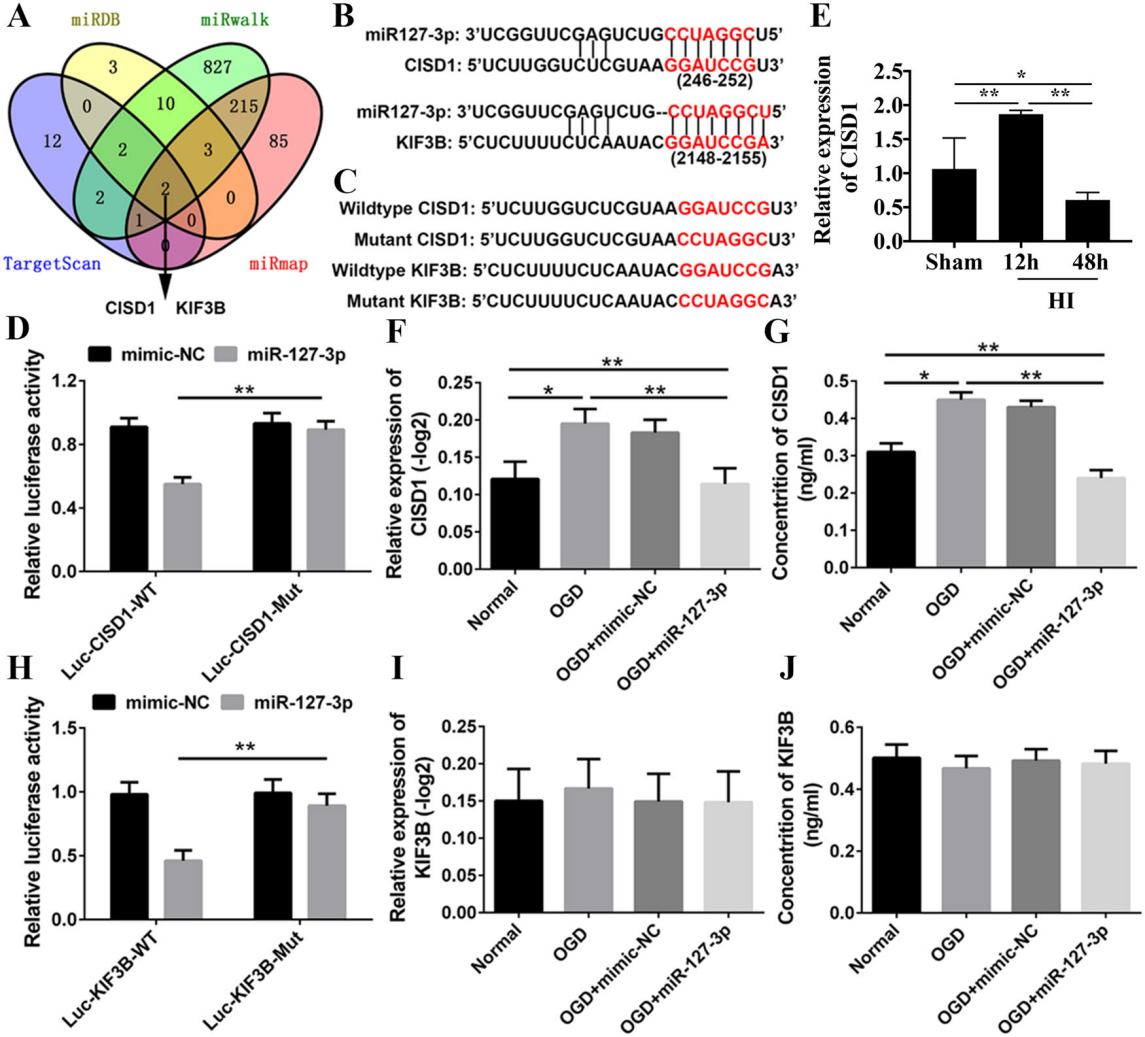
The detection of the direct targets of miR-127. **A** Venn diagram of target genes of miR-127-3p which derived from conventional online programs of miRDB, TargetScan, miRwalk and miRmap. CISD1 and KIF3B are the common targets of miR-127-3p in the four conventional online programs which were selected for further investigation. **B** The alignment of the seed regions of miR-127-3p with CISD1 and KIF3B. **C** The wildtype and mutation alignment sequence of CISD1 and KIF3B. **D** Luciferase activity of CISD1 was detected at 48 h after transfection. Relative luciferase activity was calculated with (RlucmiRNA/hLuc miRNA)/(Rluc NS-miRNA/hLuc NS-miRNA). **E** qRT-PCR analysis of CISD1 expression at 12 and 48 h after HI injury. **F**, **G** The relative expression and concentration of CISD1 were detected. **H** Luciferase activity of KIF3B. **I**, **J** The relative expression and concentration of KIF3B were detected. *P < 0.05, **P < 0.01 with one-way ANOVA, *n* = 6.

To determine the function of miR-127-3p in vivo, KO-miR-127-3p SD rats were constructed by using short palindromic repeats (CRISPR)/Cas9 (Fig. 3A, B). The results of TTC staining and quantitative analysis revealed a significant decrease of percentage of infarct volume in miR-127 KO rats (Fig. 3C, D, *P* < 0.01). Moreover, neurological function and behavioral cognition of miR-127 KO rats were notably improved according to the results of Zea-longa score (Fig. 3E, *P* < 0.001), body weight (Fig. 3F, *P* < 0.01), gait test (Fig. 3G, *P* < 0.001), righting reflex test (Fig. 3H, *P* < 0.001), rote rod test (Fig. 3I, *P* < 0.001), crossing test (Fig. 5J, *P* < 0.01), and latency to platform (Fig. 3K, *P* < 0.01). In addition, the autophagosomes were disappeared in miR-127 KO group, when compared with neonatal HI group (Fig. 3L, *P* < 0.01). The right cerebral blood flow was dramatic improvement of KO-miR-127-3p from sham and HI group (Fig. 3M, N, *P* < 0.01). Moreover, the expression of the main autophagy-related proteins, P62, ATG12, Beclin-1, and LC3II, was markedly decreased in KO-miR-127-3p group (Fig. 3O–R, *P* < 0.01), when compared with HI group. Meanwhile, autophagy of neurons was increased significantly in OGD, and reduced obviously in miR-127-3p inhibitor (Fig. 4A). After OGD, the relative expression of ATG12, Beclin-1, P62, and LC3II has shown an obvious increase, while the relative expression of LC3I was decreased significantly. However, the expression results of ATG12, Beclin-1, P62, LC3I, and LC3II were opposite in the miR-127 KO group (Fig. 4B–G). As the accumulation of autophagy-related proteins provided an effective way of detecting autophagosomes, the results illuminated that KO-miR-127-3p largely restrained germination of autophagy in the cerebral cortex of neonatal HI rats, which was closely related to the neuroprotection of KO-miR-127-3p.

### miR-127-3p directly regulated the expression of CISD1

miR-127-3p target gene in primary cortical neurons was searched. CISD1 and KIF3B were identified by four Bioinformatics databases containing miRDB (http://www.mirdb.org/), Target Scan (http://www.targetscan.org/vert_72/), miRNA Walk (http://zmf.umm.uniheidelberg.de/apps/zmf/mirwalk2/), and miRmap (http://mirmap.ezlab.org/app/) (Fig. 5A). The 3′-UTRs of CISD1 and KIF3B were cloned into the pmi-R-RBREPORT plasmid, and the predictions of binding sites of miR-127-3p were as shown in the figure (Fig. 5B). The mutation structure was generated by using the reporter gene site-directed mutation containing CISD1 and KIF3 gene 3′-UTRs (Fig. 5C). In order to reduce the luciferase activity of CISD1 cells transfected with 3′-UTRs, miR-127-3p was administered, however, it did not play a role in the cells transfected plasmid containing the mutant 3′-UTRs of CISD1 (Fig. 5D, *P* < 0.01). Besides, the results of qRT-PCR in HI group showed that the expression of CISD1 was obviously increased at 12 h, followed by decreased at 48 h after HI (Fig. 5E, *P* < 0.01). To identify whether miR-127-3p was involved in the regulation of CISD1, we confirmed that addition of miR-127-3p mimic altered the expression of endogenous CISD1. Moreover, miR-127-3p mimic administration effectively suppressed the expression of CISD1 (Fig. 5F, G, *P* < 0.01). After cells were transfected with plasmids containing 3′-UTRs of KIF3, the luciferase activity was largely decreased in miR-127-3p, and the inhibitory effect was abolished in the plasmid containing the mutant 3′-UTRs of KIF3 (Fig. 5H, *P* < 0.01). However, the expression of KIF3 showed no obvious changes by the administration of miR-127-3p mimic (Fig. 5I, J, *P* < 0.01). In summary, the results suggested that CISD1 was a major direct target of miR-127-3p in neurons.

### CISD1 overexpression alleviated mitochondrial function and cortical neuron injury induced by OGD in vitro

**Fig. 6:**
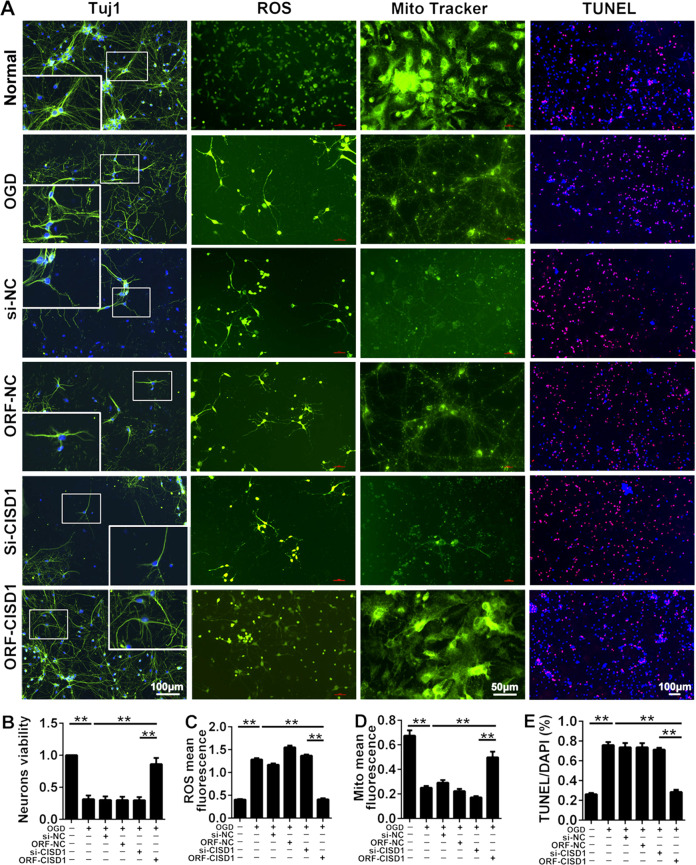
The effects of CISD1 overexpression on mitochondrial function and neuron injury induced by OGD in vitro. **A** Neurons were stained by Tuj1, ROS, Mito tracker, and TUNEL to detect the condition of neurons, the level of ROS, the condition of mitochondria and neurons apoptosis in normal group, OGD group, si-NC group, ORF-NC group, si-*CISD1*, and ORF-*CISD1* group. Scale bar = 100 μm (Mito tracker: scale bar = 50 μm). **B** The bar charts of neurons viability in neurons of each group. Compared with normal group, the neurons viability decreased in OGD group. While the neurons viability increased in ORF-CISD1 group, compared with OGD group. **C** The level of ROS in neurons of each group. ROS significantly increased in OGD group, compared with the normal group. Whereas ROS decreased significantly in ORF-CISD1 group, compared with OGD group. **D** The mitochondria mean fluorescence in neurons of each group. Mito mean fluorescence significantly decreased in OGD group, compared with normal group. Whereas mito mean fluorescence significantly increased in ORF-CISD1 group, compared with OGD group. **E** Percentage of TUNEL/DAPI of neurons was shown in each group. TUNEL staining was used to analyze neuronal apoptosis. The percentage of TUNEL/DAPI significantly increased in OGD group, compared with normal group. Whereas percentage of TUNEL/DAPI significantly decreased in ORF-CISD1 group, compared with OGD group. The data were presented as the mean ± s.d. **P < 0.01 with one-way ANOVA, *n* = 6. *CISD1* CDGSH iron sulfurdomain-containing protein 1, OGD oxygen glucose deprivation, HI hypoxia ischemia, ROS reactive oxygen species, ORF-*CISD1* *CISD1* overexpression, si-*CISD1* *CISD1* low expression, ORF-NC negative control overexpression, si-NC negative control low expression, Tuj1 neuronal class III β-tubulin, TUNEL terminal deoxynucleotidyl transferase dUTP nick end labeling.

**Fig. 7:**
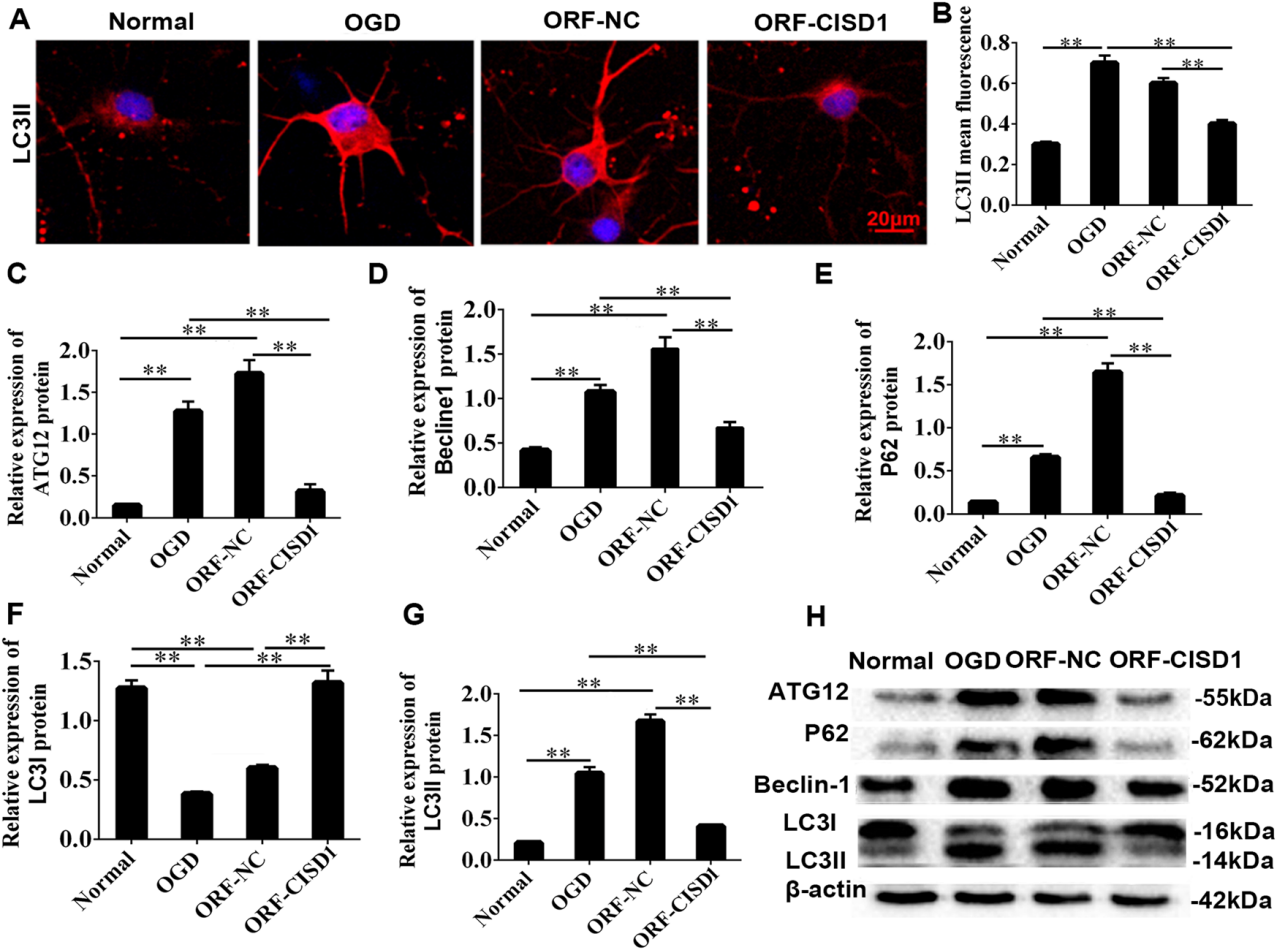
CISD1 relieved autophagy in vitro. **A** Autophagy of neurons was increased significantly in OGD, and reduced significantly in ORF-CISD1. **B** The relative expression of LC 3II was obviously increased in OGD, but reduced significantly in ORF-CISD1. OGD oxygen glucose deprivation. **C**–**H** The relative expression of ATG12, Beclin-1, P62, and LC 3II was obviously increased, and the relative expression of LC 3I was decreased significantly. However, the expression result of ATG12, Beclin-1, P62, LC 3I, and LC 3II was opposite in the miR127 KO rat group. Scale bar = 20 μm. Data are shown as mean ± s.d. **P < 0.01 with one-way ANOVA or Kruskal–Wallis, *n* = 6. OGD oxygen glucose deprivation, NC negative control, CISD1 CDGSH iron sulfurdomain-containing protein 1, ORF-CISD1 CISD1 overexpression, CISD1-NC negative control CISD1.

ORF-CISD1 lentivirus and si-CISD1 lentivirus were constructed to validate the function of CISD1 in PC12 cells, SY5Y cells and neuron after OGD. Results showed OGD administration overtly reduced the number of the three kinds of cells compared with sham group, while ORF-CISD1 significantly increased the cells compared with OGD group (Fig. S2, *P* < 0.01). Between si-CISD1 group and OGD group, there were no obvious difference from three kinds cells. These results suggested CISD1 overexpression evidently improved the cell damage caused by OGD. In order to further explore the function of CISD1, the morphologies of neurons, level of ROS, mitochondrial activity, and apoptosis in cortical neurons were detected (Fig. 6A). Comparison with the results of the inhibition of CISD1, the CISD1 overexpression ameliorated neuronal viability significantly, inhibited the formation of ROS in cortical neurons, improved the mitochondrial activity, and attenuated neuronal apoptosis (Fig. 6B–E, *P* < 0.01). The morphology of neurons was stained with immunofluorescence, the fluorescence of LC3II was obviously increased in OGD, then it was decreased in CISD1 overexpression (Fig. 7A, B, *P* < 0.01). In addition to LC3I protein, the expression of autophagy-related proteins ATG12, P62, Beclin-1, and LC3II were significantly increased. Meanwhile, CISD1 overexpression could reverse this upward trend (Fig. 7C–H, *P* < 0.01). Therefore, CISD1 overexpression could alleviate mitochondrial function and cortical neuron injury induced by OGD in vitro.

### Overexpression of CISD1 inhibited autophagy and neurological impairment in vivo

**Fig. 8:**
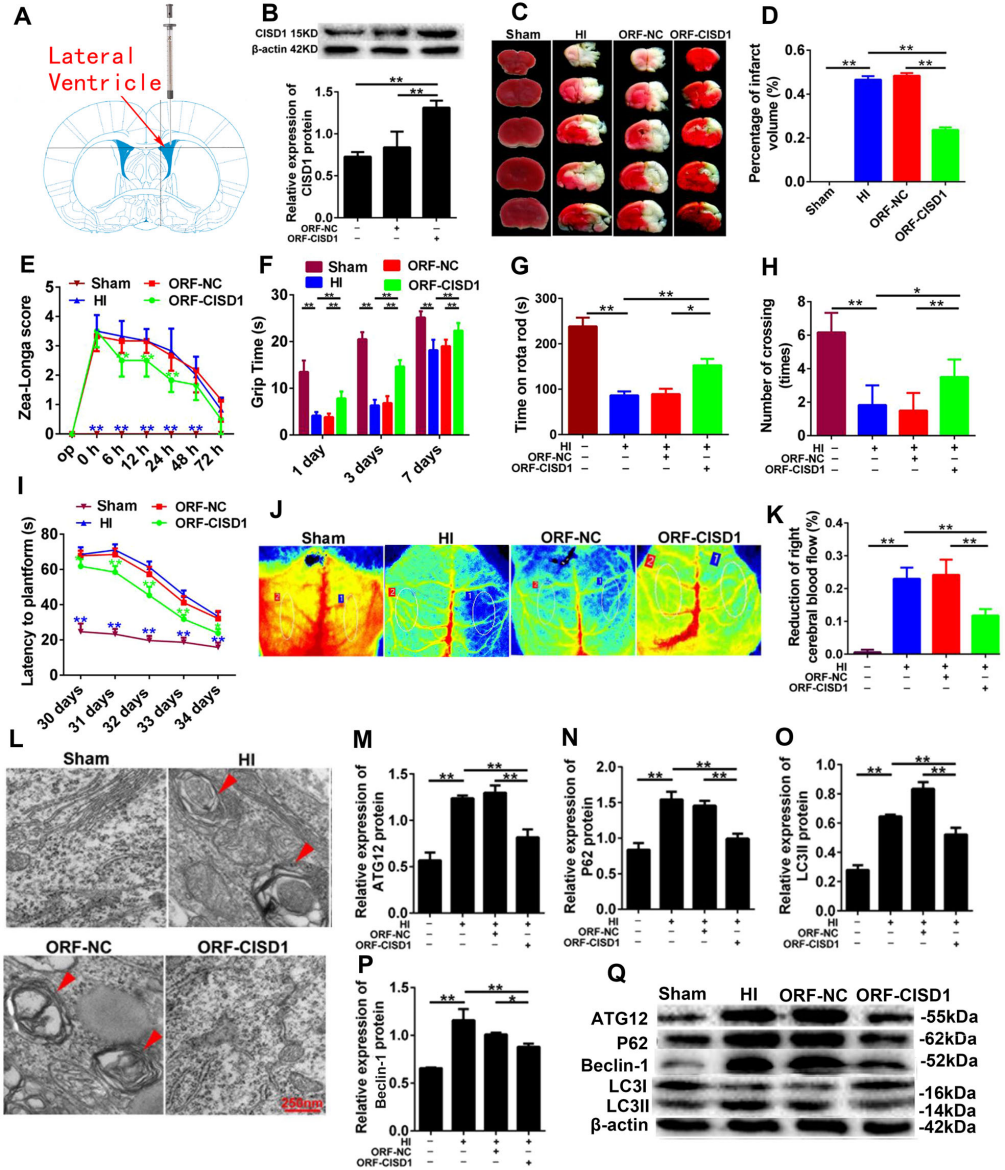
The role of CISD1 overexpression in autophagy and neurological impairment in vivo. **A** The ideograph of ORF-*CISD1* lentivirus injection site. **B** The protein expression of *CISD1* increased after injection of ORF-*CISD1* lentivirus. **C** TTC staining of rat brains. Scale bar = 1 cm. **D** Infarcts were quantified by planimetry and expressed as a percentage of risk zones. **E** Zea-longa score of HI rats. **F** Grip times were assessed in 1 d, 3 d, and 7 d after HI. **G** Time on rotarod was assessed in 38 d after HI. **H** Number of crossing was recorded in 38 d after HI. **I** Latency to platforms were assessed in 30 d, 31 d, 32 d, 33 d, and 34 d after HI. **J**, **K** Reduction of right cerebral blood flow after HI. **L** Representative EM images of autophagic vacuoles were shown. Scale bar = 250 nm. The arrows depict autophagosomes. **M**–**Q** The expression of ATG12, P62, Beclin-1 LC3I, and LC3II were detected by western blotting. Representative immunoblot for conversion of LC3I to LC3II in Sham group, HI group, ORF-NC group and ORF-*CISD1* group. The densitometric analysis of WB of LC3II is shown. Data are shown as mean ± s.d. *P < 0.05, **P < 0.01 with one-way ANOVA, *n* = 6. *CISD1* CDGSH iron-sulfur domain-containing protein 1, HI hypoxia ischemia, ORF-NC negative control overexpression, ORF*CISD1 CISD1* overexpression, H hours, S seconds.

The ORF-CISD1 *lentivirus* (LV) were injected into the neonatal rats subjected to neonatal HI (Fig. 8A), and it was found that the expression of CISD1 protein was obviously increased in the cerebral cortex (Fig. 8B, *P* < 0.01). The result manifested that in morphology and neurological function, the infarct volume, Zea-longa score and latency to platforms in ORF-CISD1 rats showed a remarkable decreased, while, the grip time, time on rota rod and number of crossing were significantly increased (Fig. 8C–I, *P* < 0.05, *P* < 0.01). Comparatively, CISD1 overexpression showed a reduction of right cerebral blood flow (Fig. 8J, K, *P* < 0.05, *P* < 0.01) and the formation of autophagosomes in the cortex of HI rats (Fig. 8L), which illuminated that the CISDI overexpression inhibited the occurrence of mitochondrial autophagy through inhibiting the generation of ROS. Additionally, the CISD1 overexpression significantly decreased the expression of autophagy associated proteins ATG12, P62, Beclin-1, LC3I, and LC3II (Fig. 8M–Q, *P* < 0.01). Together, the overexpression of CISD1 could evidently ameliorate the incidence of autophagic apoptosis in the cortical neurons of rats with HI injury.

### The neuroprotection of KO-miR-127-3p was induced by upregulating CISD1 in vitro

KO-miR-127-3p administration obviously attenuated neurons damage after OGD (Figs. S3, S4A, and S5A), and KO-miR-127-3p markedly improved the neuronal activity, mitochondrial activity, and decreased the value of fluorescence in LC3II, obviously, while they were reversed in KO-miR-127-3p together with si-CISD1 group (Fig. S4B, *P* < 0.01). Meanwhile, KO-miR-127-3p could reduce the expression of autophagy-associated proteins of ATG12, P62, Beclin1, and LC3II, while, si-CISD1 showed the opposite effect (Fig. S5B–G, *P* < 0.01). Moreover, co-inhibition of miR-127-3p and CISD1 reduced significantly neuronal activity, decreased the mitochondrial activity, and increased the neurons apoptosis and the level of ROS, confirming that the neuroprotection of KO-miR-127-3p was realized by increasing CISD1 expression (Fig. S6, *P* < 0.01).

### KO-miR-127-3p inhibited autophagy and improved neurological impairment through upregulating the expression of CISD1 in vivo

In vivo, si-CISD1 was injected lentivirus into the neonatal rats treated with KO-miR-127-3p to detect the changes of autophagy and neuropathology. Inhibition of miR-127-3p overtly reduced the formation of autophagosome and downregulated the expression of autophagy-associated proteins of ATG12, P62, Beclin1 and LC3II, while the expression of these proteins was greatly increased and autophagosome appeared after inhibiting miR-127-3p and CISD1 simultaneously (Fig. S7A–E, *P* < 0.01). Meanwhile, KO-miR-127-3p and CISD1 overexpression had the same neuroprotective effect, which was achieved by inhibiting the generation of ROS, improving neuronal mitochondrial activity, and inhibiting neural autophagy. In morphology, inhibition of CISD1 significantly reversed the reduction of infarct area in KO-miR-127-3p rats (Fig. S7F, G, *P* < 0.01). Additionally, the neurologic function of neonatal SD rats was detected by rota rod test (Fig. S7H, **P < 0.01), grip time test (Fig. S7I, *P* < 0.01), showed that the inhibition of CISD1 aggravated the cortical neurologic function in KO-miR-127-3p rats. Together, these data validated that KO-miR-127-3p inhibits autophagy and cortical neurological impairment through upregulating the expression of CISD1 in rats with HI injury.

## Discussion

Sequestration of miR-127-3p have relieved autophagic apoptosis and exerted a neuroprotection in the cortex of neonatal rats with neonatal HI injury, and the underlying mechanism is involved with CISD1 overexpression. Our study provided a direct evidence to understand miR-127-3p targeting CISD1 to affect autophagy in neonatal rats subjected to HI injury.

### Role of miR-127-3p in HI

#### Screen of miR-127-3p

In this research, we used microRNA chip technology to screen and found a variety of microRNAs with different expressions in the cerebral ischemic of newborn rats, among which miR-127-3p is a molecule with obvious downregulated expression. Moreover, the results of qRT-PCR in different organs showed miR-127-3p was specifically and highly expressed in the brain tissue, which indicated that miR-127-3p was a miRNA specifically expressed in the central nervous system. Meanwhile, the expression of miR-127-3p was visibly downregulated in PC12 cells, SY5Y cells, and cortical neurons after OGD. These results are consistent with those in our cortical tissue, which further indicates the potential research value of mir-127-3p in neonatal HI brain injury. Therefore, mir-127-3p was selected as the key molecule for further study.

#### Role of miR-127-3p in HI

At first, sequestration of miR-127-3p showed a significant inhibition for ROS generation, mitochondrial inactivation and autophagic apoptosis in cortical neurons, which underly the basis on neurological improvement in the cerebral cortex of HI. Previously, it is reported that miR-127-3p is downregulated in many cancers, including hepatocellular carcinoma, breast cancer, and oral cancers, which was able to suppress cell growth and enhanced apoptosis^27,28^. In central nervous system, miR-127-3p is a kind of miRNA enriched by neurons and plays an important role in neuron differentiation^26,29,30^. Here, we showed a new important role for miR-127-3p in neurons autophagy in HI injury. Autophagy has been reported to have an adaptive role in protecting neurons from ischemic damage^31,32^. However, the abnormal enhanced autophagy induced a variety of pathological disorders, including cancer, cardiovascular diseases, neurodegenerative, and further HI injury^33–38^. Therefore, different autophagy-related signaling pathways, as well as different concentrations or times of stimulus trigger, will determine whether autophagy is to inhibit or promote neuronal death, which needs to be depicted during neuronal ischemic injury. In recent years, increasing miRNAs were key regulators of different cellular processes^39^, specifically, related to autophagy^40,41^. However, few studies identified that these miRNAs are related to autophagy to affect neuron autophagy. In our research, we noticed that inhibition of miR-127-3p played important roles in autophagic apoptosis in neonatal cortical neurons after HI.

### Role of CISD1 in HI

In our experiment, it was discovered that inhibiting mir-127-3p can improve the nerve function of rats with hypoxic ischemic brain injury, and this neuroprotective effect is exerted by reducing the generation of reactive oxygen species in hypoxic ischemic neurons, improving mitochondrial activity, and thereby reducing the mitochondrial autophagy of neurons. CISD1 and KIF3B, two potential target genes of mir-127-3p, were found through the analysis of four miRNAs target gene prediction bioinformatics databases: miRDB, TargetScan, miRNAWalk, and miRmap. Among them, CISD1 is a mitochondrial membrane protein and contains the iron ion transport domain, which plays an important role in the regulation of mitochondrial iron ion transport and oxidative stress, and is closely related to the HI of neurons after ischemia. Then we used Luciferase and mutant studies to find that the direct target of miR-127-3p was the base sequence GGAUCCG at CISD1 5 ‘end 246-252. Moreover, after transfection with the agonist and inhibitor of miR-127-3p in cortical neurons, it was found that CISD1 protein expression was significantly reduced in neurons after miR-127-3p was overexpressed, while CISD1 protein expression was significantly increased in neurons after miR-127-3p was inhibited.

### miR-127-3p targeting CISD1 in HI

Moreover, in this study, miR-127-3p and CISD1 showed negative correlated modulators. Mechanistic evidence of CISD1 overexpression played a primary role in neuroprotection of miR-127-3p sequestration observed in the cerebral cortex of neonatal HI injury. CISD1 could associate the homeostasis of iron and ROS in neurons with mitochondrial function, which associated with several human diseases, including diabetes, that are associated with this type of iron-sulfur proteins, Wolfram syndrome 2, and neurodegeneration^42,43^. The lack of CISD1 results in the accumulation of iron and ROS in mitochondria of animal cells, the decline of mitochondrial function and stability in mice and human cells, and the activation of autophagy^44^. In this study, it has been pointed that CISD1 was a major target of miR-127-3p in cortical neurons, which also affected ROS generation, mitochondrial activation, and cerebral ischemic injury. Moreover, the neuroprotection of KO-miR-127-3p or CISD1 overexpression was related to inhibition of autophagy. Therefore, KO-miR-127-3p can inhibit autophagy and cerebral infarction by upregulating CISD1 protein. In other words, miR-127-3p exploits a new mechanism to regulate CISD1 expression to drive autophagy.

## Conclusion

Together, our results revealed that the knockdown of miR-127-3p activate autophagy in the cortical neurons by targeting CISD1 overexpression in neonatal rats with neonatal HI injury. As a result, our data provided the evidence to demonstrate the mechanism of HI injury, in which, miR-127-3p targeting CISD1 is core mechanism to regulate autophagy in neonatal HI cortex. Moreover, miR-127-3p and/or CISD1 could be considered as potential targets for the therapy of neonatal HI brain injury in future clinic practice.

## Supplementary information

Supplementary Figure Legends

Supplementary figure1

Supplementary figure2

Supplementary figure4

Supplementary figure5

Supplementary figure6

Supplementary figure7

Supplementary figure3

## Data Availability

The datasets generated and analyzed in the current study can be obtained from Pro. Ting-Hua Wang’s Laboratory.
