# Supplementary material for: MiR-127-3p targeting CISD1 regulates autophagy in hypoxic–ischemic cortex
Source: Cell Death Dis. 2021 Mar 15;12(3):279. doi: 10.1038/s41419-021-03541-x (PMC7961148; doi:10.1038/s41419-021-03541-x)
Supplement: Supplementary file 1 — Supplementary Figure Legends [file 41419_2021_3541_MOESM1_ESM.doc]

**Supplementary Figures:**

***Fig. S1 The effects of miR-127-3p inhibition on PC12 cells, SY5Y cells and neurons after OGD.***

(A) The changes of cells number in normal group, OGD group, mimic-NC group, anti-NC group, miR-127-3p group and anti-miR-127-3p group were showed by bright field image. Experiment was carried on PC12 cells, SY5Y cells and neurons at the same time and condition. Scale bar=50 μm. (B) The average number of PC12 cells in each group. The number of PC12 cells significantly decreased in OGD group, compared with normal group. While the number of PC12 cells increased in anti-miR-127-3p group, compared with OGD group. (C) The average number of SY5Y cells. The number of SY5Y cells significantly decreased in OGD group, compared with normal group. While the number of SY5Y cells significantly increased in anti-miR-127-3p group, compared with OGD group. (D) The average number of neurons. The number of neurons significantly decreased in OGD group, compared with normal group. While the number of neurons significantly increased in anti-miR-127-3p group, compared with OGD group. The data were presented as the means ± s.d. ***P* < 0.01 with one-way ANOVA, n = 6. MiR: MicroRNA. OGD: oxygen glucose deprivation. NC: Negative Control.

***Fig. S2 The effects of CISD1 on the reduction of PC12 cells, SY5Y cells and neurons after OGD.***

(A) The changes of cells number in normal group, OGD group, si-NC group, ORF-NC group, si-CISD1 group and ORF-CISD1 group were showed by bright field imaging. Experiment was carried on PC12 cells, SY5Y cells and neurons at the same time and condition. Scale bar=50 μm. (B) The average number of PC12 cells in each group. The number of PC12 cells significantly decreased in OGD group, compared with normal group. While the number of PC12 cells increased in ORF-CISD1 group, compared with OGD group. (C) The average number of SY5Y cells. The number of SY5Y cells significantly decreased in OGD group, compared with normal group. While the number of SY5Y cells significantly increased in ORF-CISD1 group, compared with OGD group. (D) The average number of neurons. The number of neurons significantly decreased in OGD group, compared with normal group. While the number of neurons significantly increased in ORF-CISD1 group, compared with OGD group. The data were presented as the means± s.d. ***P* < 0.01 with one-way ANOVA, n = 6. si-NC: Negative Control low expression. si-*CISD1*: *CISD1* low expression. ORF-NC: Negative Control overexpression. ORF-*CISD1*: *CISD1* overexpression. OGD: oxygen glucose deprivation.

***Fig. S3 The role of KO-miR-127-3p in neurons injury after OGD by targeting CISD1.***

1. The change of neuron number was showed by light microscope in normal group, OGD group, KO-miR-127-3p group, KO-miR-127-3p + si-NC group and KO-miR-127-3p + si-CISD1 group. Scale bar=50 μm. (B) The bar chart of average number of neurons. Compared with normal group, the number of neurons decreased in OGD group; Compared with OGD group, the number of neurons increased in KO-miR-127-3p group; Compared with KO-miR-127-3p + si-NC group, the number of neurons decreased in KO-miR-127-3p + si-CISD1 group. The data were presented as the means± s.d. **P* < 0.05, ***P* < 0.01 with one-way ANOVA, n = 6. si-NC: Negative Control low expression. si-*CISD1*: *CISD1* low expression. KO: Knock-out. OGD: oxygen glucose deprivation.

**Figure S4. The role of KO-miR-127-3p in neurons injury after OGD by targeting CISD1.**

1. Double labeling of immunofluorescence in neurons injury. The change of neuron number was showed by light microscope in normal group, OGD group, KO-miR-127-3p group, KO-miR-127-3p + si-NC group and KO-miR-127-3p + si-CISD1 group. Scale bar=20 μm. (B) The relative expression of LC 3Ⅱ were obviously increased in OGD, but reduced significantly in KO-miR-127-3p and increased in KO-miR-127-3p + si-CISD1 group. si-NC: Negative Control low expression. si-CISD1: CISD1 low expression. The data were presented as the means± s.d. ***P* < 0.01 with one-way ANOVA, n = 6. KO: Knock-out. OGD: oxygen glucose deprivation. Tuj1: Neuronal Class III β-Tubulin. DAP1: 4',6-diamidino-2-phenylindole.

**Figure S5. The relationship of KO-miR-127-3p and CISD1 in vitro.**

1. Neurons were stained by LC3II in normal group, OGD group, KO-miR-127-3p group, KO-miR-127-3p + CISD1-NC group and KO-miR-127-3p + CISD1-ORF group. Scale bar=20 μm. (B, C, D, E, F, G) The expression of ATG12, P62, Beclin1, LC3 I and LC3II were detected by WB in Sham group, OGD group, KO-miR-127-3p group, KO-miR-127-3p + CISD-NC group and KO-miR-127-3p + si-CISD1group. The data were presented as the means± s.d. ***P* < 0.01 with one-way ANOVA or Kruskal–Wallis, n = 6. Tuj1: Neuronal Class III β-Tubulin. OGD: oxygen glucose deprivation. KO: knock out.

**Figure S6. The relationship between the neuroprotection of KO-miR-127-3p and si-CISD1 *in vitro.***

1. Neurons were stained by Tuj1, ROS, Mito tracker and TUNEL to detect the condition of neurons, the level of ROS, the condition of mitochondria and neurons apoptosis in normal group, OGD group, KO-miR-127-3p group, KO-miR-127-3p + si-NC group and KO-miR-127-3p + si-*CISD1* group. Scale bar=100 μm (Mito tracker: scale bar=50 μm). (B) The bar charts of neurons viability in neurons among normal, OGD, KO-miR-127-3p, KO-miR-127-3p + si-NC group, KO-miR-127-3p + si-*CISD1* groups. (C) The level of ROS in neurons of each group. ROS significantly increased in KO-miR-127-3p + si-*CISD1* group, compared with KO-miR-127-3p + si-NC group. (D) The mitochondria mean fluorescence in neurons of each group. Mito mean fluorescence significantly decreased in KO-miR-127-3p + si-*CISD1* group, compared with KO-miR-127-3p + si-NC group. (E) Percentage of TUNEL/DAPI of neurons was shown in each group. TUNEL staining was used to analyse neuronal apoptosis. The percentage of TUNEL/DAPI significantly increased in KO-miR-127-3p + si-CISD1 group, compared with KO-miR-127-3p + si-NC group. The data were presented as the means ± s.d. ***P* < 0.01 with one-way ANOVA, n = 6. si-*CISD1*: *CISD1* low expression. si-NC: negative control low expression. Tuj1: Neuronal Class III β-Tubulin. TUNEL: Terminal deoxynucleotidyl transferase dUTP nick end labelling. ROS: reactive oxygen species. OGD: oxygen glucose deprivation. KO: knock out.

**Figure S7. KO-miR-127-3p inhibits autophagy and neurological impairment through CISD1 *in*** ***vivo.***

(A) Representative EM images of autophagic vacuoles were shown. Scale bar=250 nm. The arrows depict autophagosomes. (B, C, D, E) The expression of ATG12, P62, Beclin1 and LC3I to LC3II were detected by WB in Sham group, HI group, KO-miR-127-3p group, KO-miR-127-3p + si-NC group and KO-miR-127-3p + si-CISD1group. (F) TTC staining of rat brains. Images above the graph shows representative slices from each group. Brains were sliced, stained with TTC and fixed to delineate live (red) from dead or infarcted tissue (white). (G) Infarcts were quantified by planimetry and expressed as a percentage of risk zone. (H) Time on rota rod was assessed in 38 d after HI. (I) Grip times were assessed in 1 d, 3 d and 7d after HI. Neurological impairment of HI rats was improved with treatment of KO-miR-127-3p when compared with HI group. Whereas, the neuro protectant of KO-miR-127-3p was inhibited by CISD1. The data were presented as the means ± s.d. **P* < 0.05, ***P* < 0.01 with one-way ANOVA or Kruskal–Wallis, n = 6. HI: hypoxia ischemia. si-*CISD1*: *CISD1* low expression. si-NC: negative control low expression. KO: knock out. S: seconds. TTC: Triphenyl tetrazolium chloride. WB: Western Bolt.
