# Supplementary figures and images for: MiR-127-3p targeting CISD1 regulates autophagy in hypoxic–ischemic cortex
Source: Cell Death Dis. 2021 Mar 15;12(3):279. doi: 10.1038/s41419-021-03541-x (PMC7961148; doi:10.1038/s41419-021-03541-x)

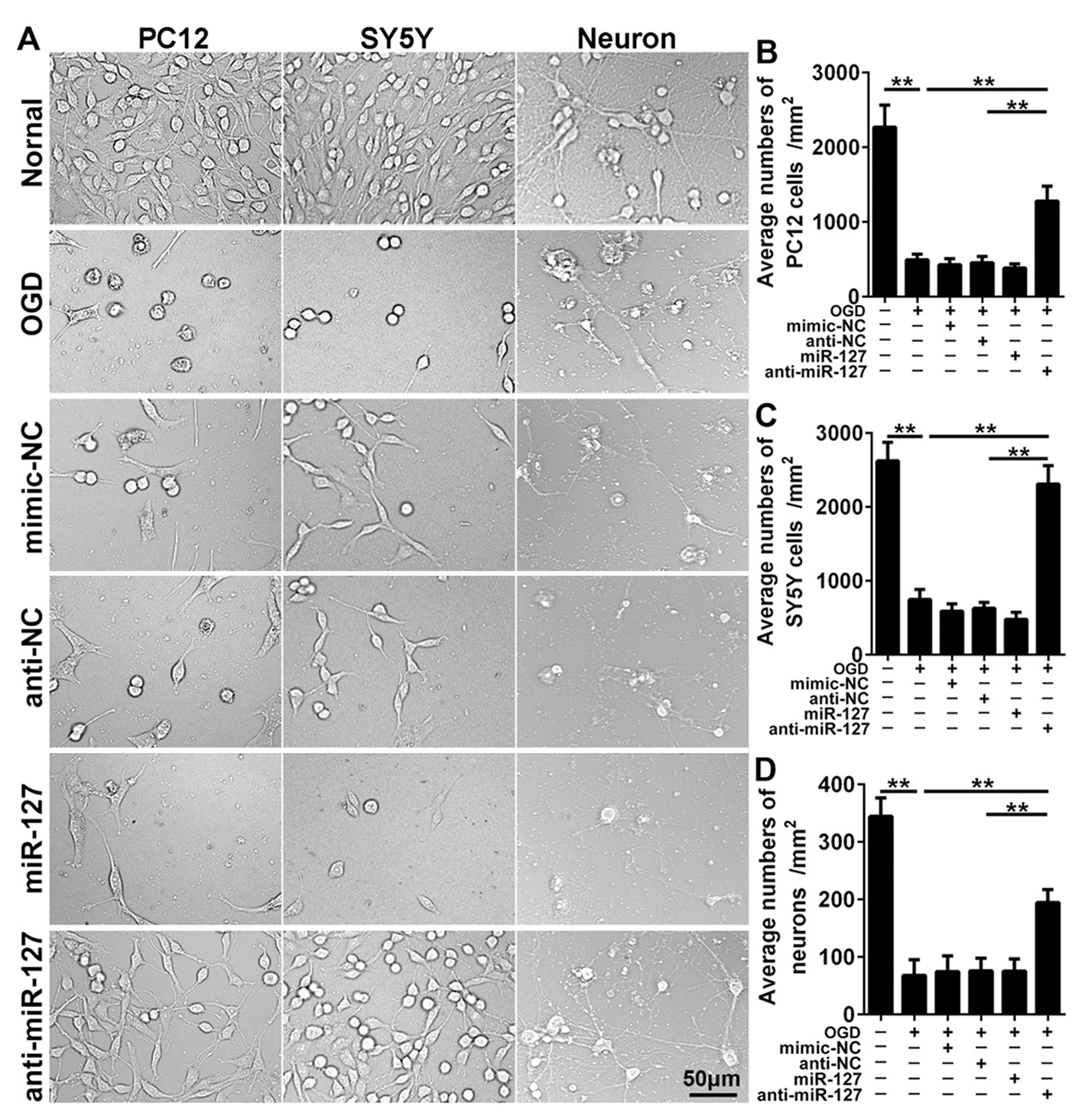

Supplement: Supplementary file 2 — Supplementary figure1 [file 41419_2021_3541_MOESM2_ESM.tif]

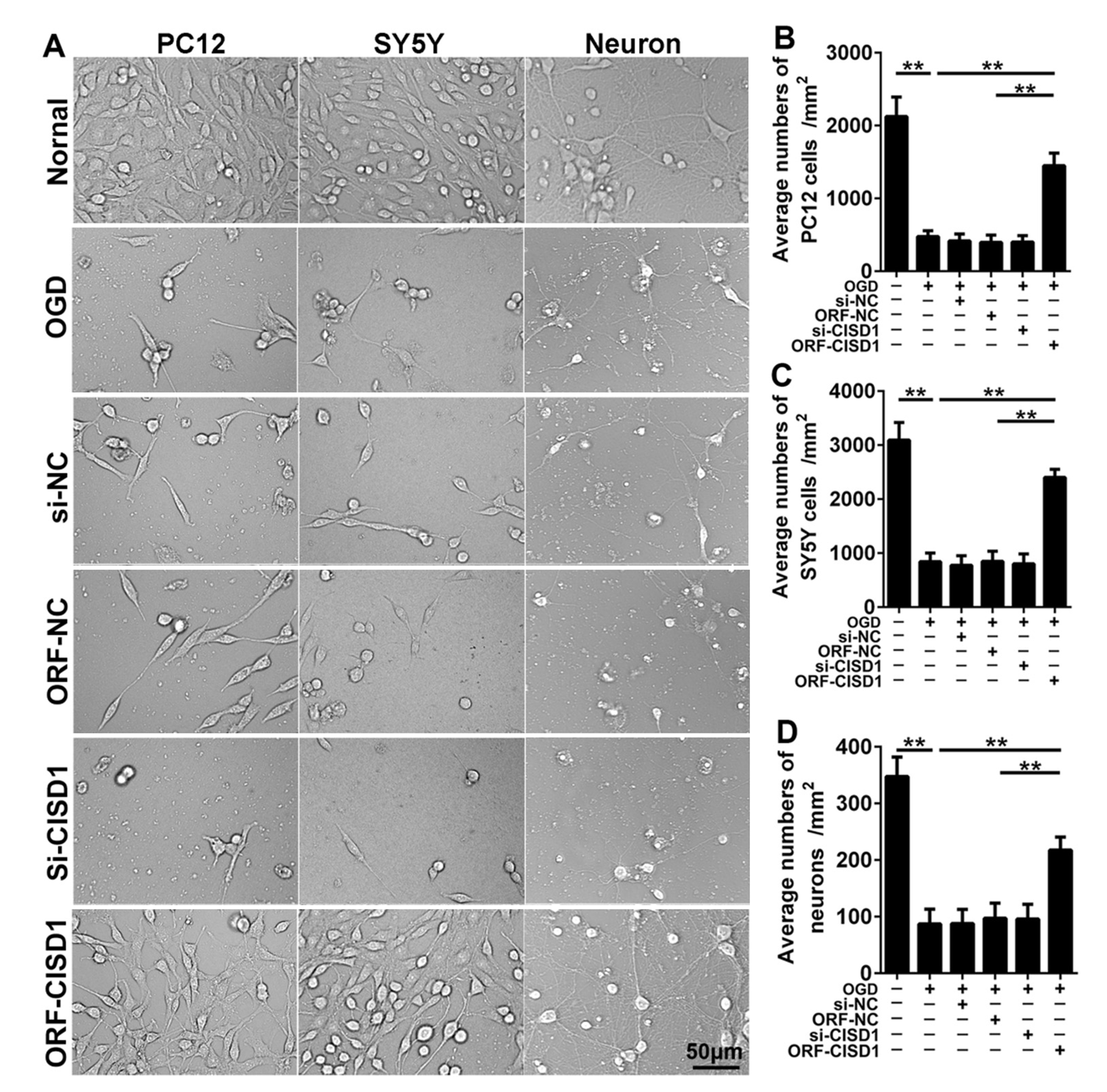

Supplement: Supplementary file 3 — Supplementary figure2 [file 41419_2021_3541_MOESM3_ESM.tif]

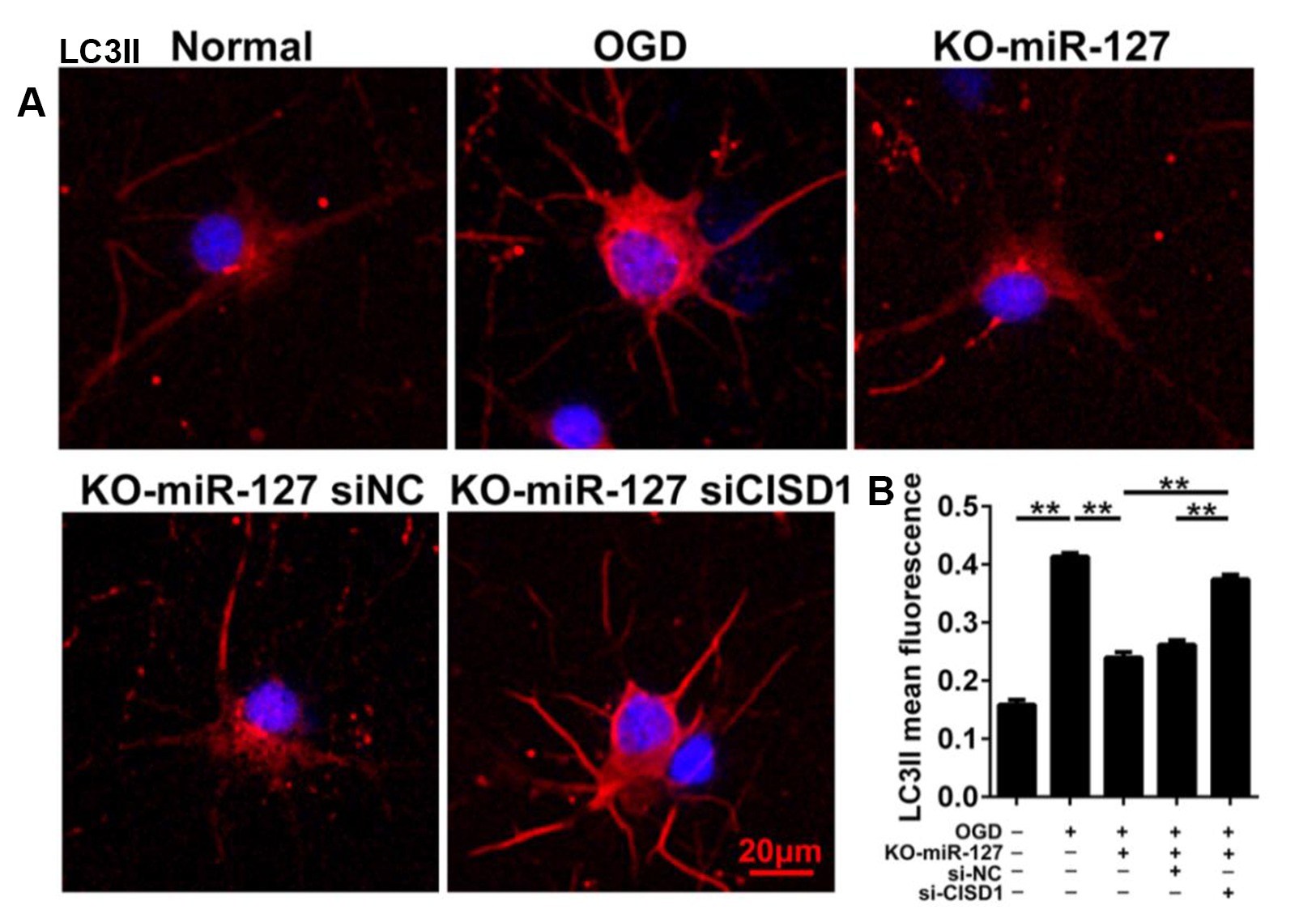

Supplement: Supplementary file 4 — Supplementary figure4 [file 41419_2021_3541_MOESM4_ESM.tif]

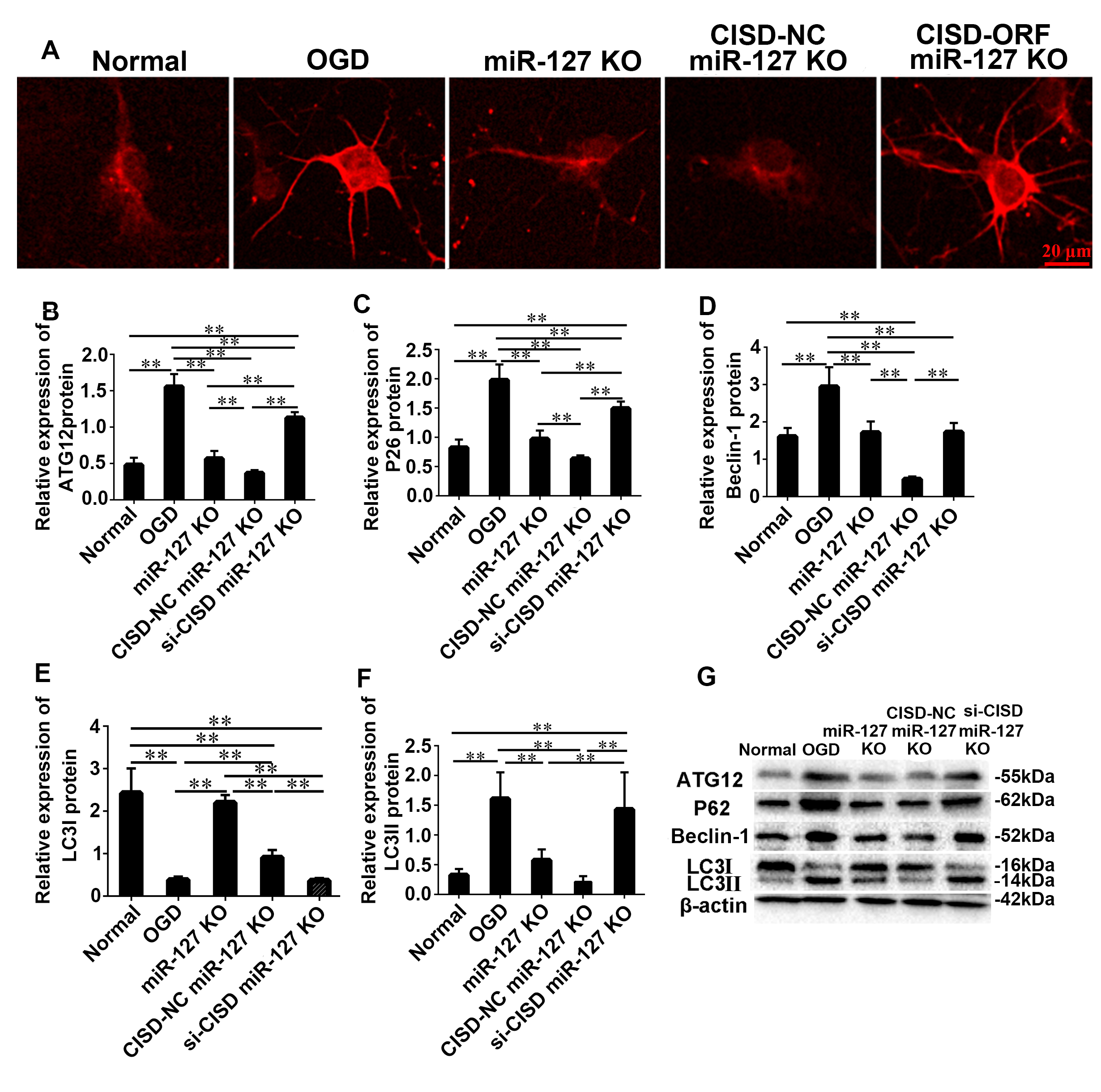

Supplement: Supplementary file 5 — Supplementary figure5 [file 41419_2021_3541_MOESM5_ESM.tif]

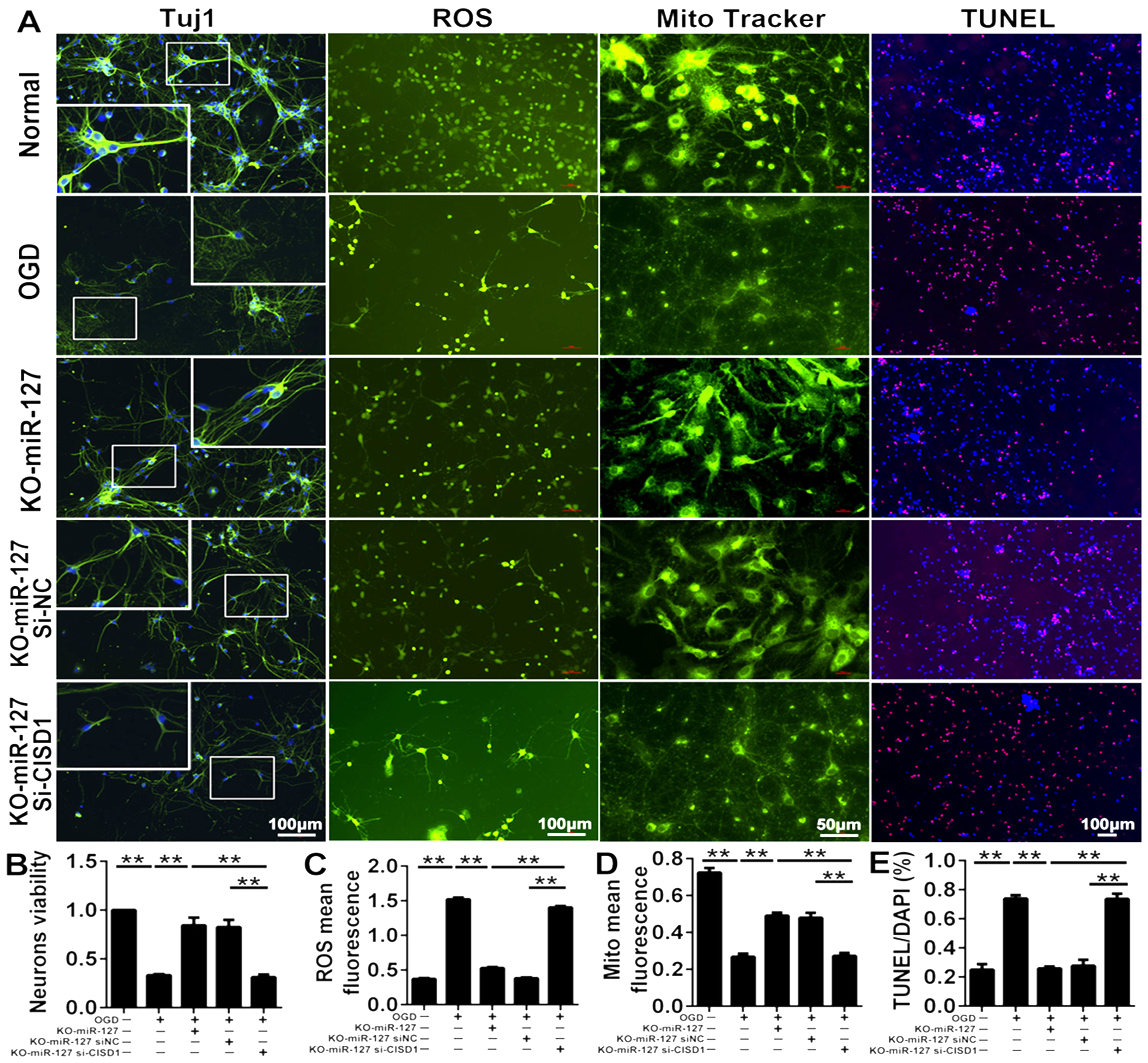

Supplement: Supplementary file 6 — Supplementary figure6 [file 41419_2021_3541_MOESM6_ESM.tif]

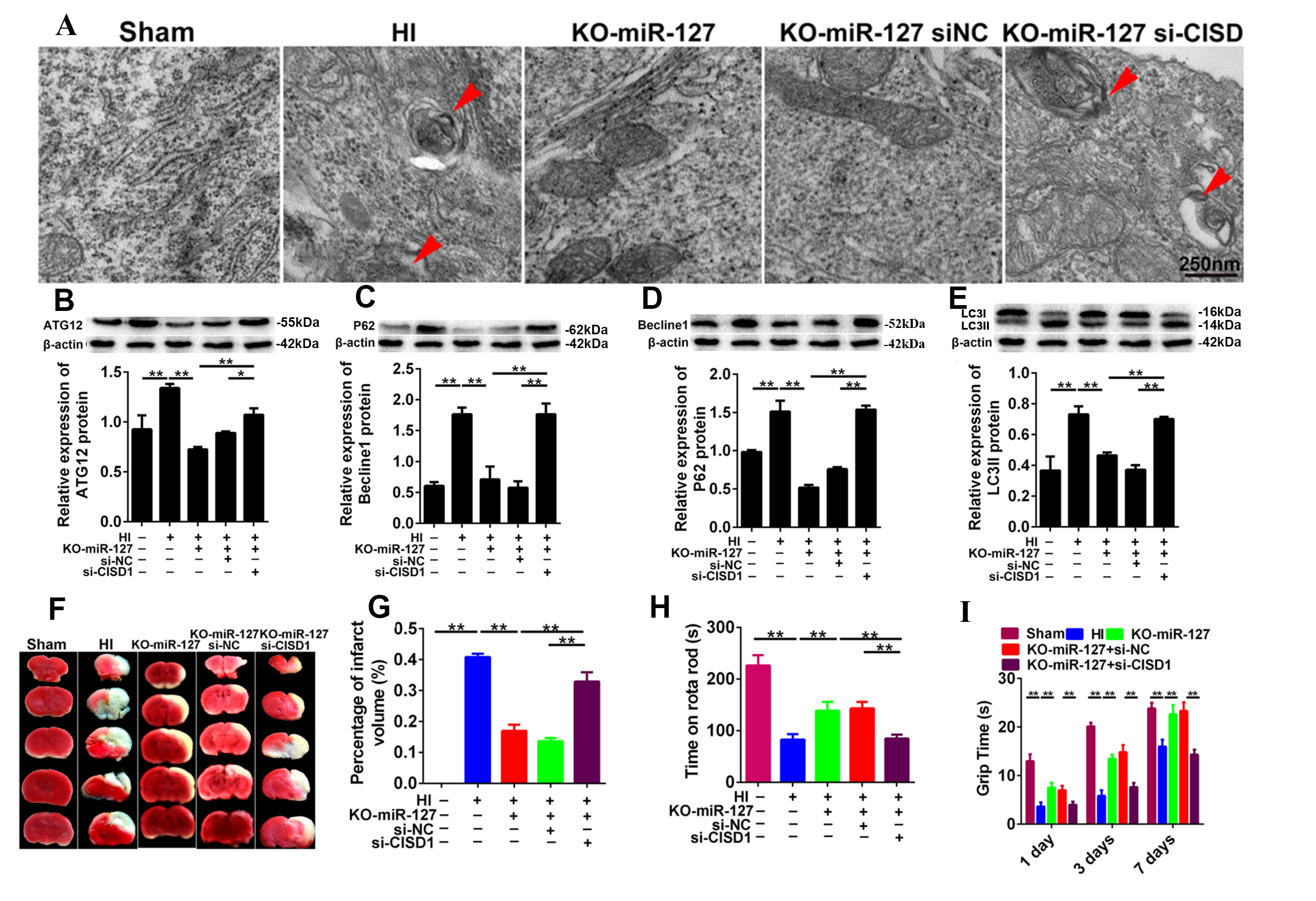

Supplement: Supplementary file 7 — Supplementary figure7 [file 41419_2021_3541_MOESM7_ESM.tif]

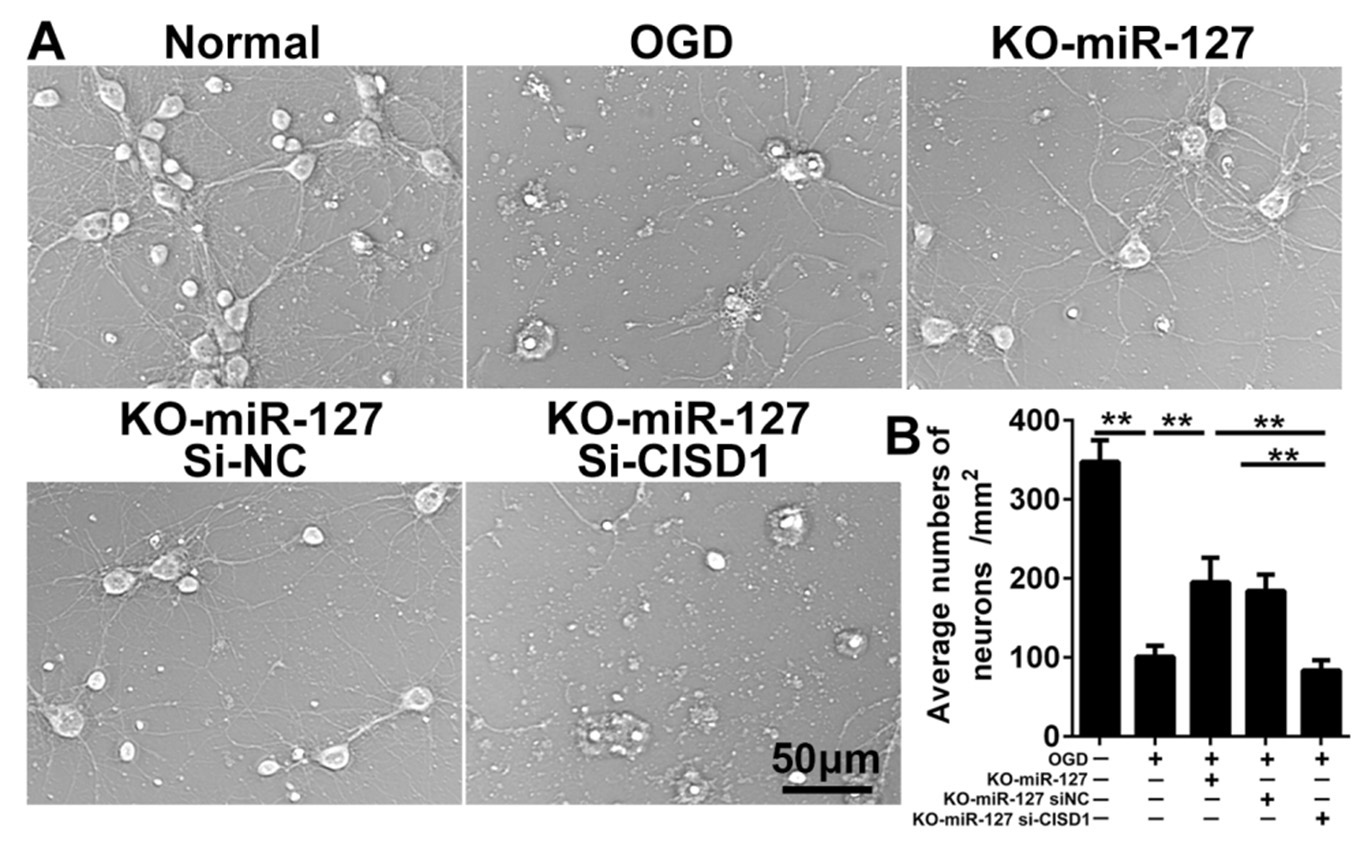

Supplement: Supplementary file 8 — Supplementary figure3 [file 41419_2021_3541_MOESM8_ESM.jpg]
